# Climate benefits from establishing marine protected areas targeted at blue carbon solutions

**DOI:** 10.1073/pnas.2121705119

**Published:** 2022-06-02

**Authors:** Emilia Jankowska, Robin Pelc, Jimena Alvarez, Mamta Mehra, Chad J. Frischmann

**Affiliations:** ^a^Drawdown Solutions, Project Drawdown, San Francisco, CA 94118;; ^b^Department of Marine Science, California State University, Monterey Bay, CA 93955;; ^c^Lancaster University Management School, Lancaster University, Lancaster LA1 4YX, United Kingdom;; ^d^Smith School of Enterprise and the Environment, University of Oxford, Oxford OX1 3QY, United Kingdom

**Keywords:** marine protected areas, blue carbon, climate change solutions, GHG, “drawdown”

## Abstract

Marine protected areas (MPAs) are recognized as highly effective tools for marine conservation. They may also play an important role in mitigating climate change. A variety of climate change solutions are rooted in the ocean, centered primarily around “blue carbon” and the capacity of marine life to sequester carbon dioxide (CO_2_) with some potential to reduce emissions. However, the global potential of these solutions remains misunderstood and untapped. Here, we analyze the potential impact on carbon removal and emissions reduction of adopting six ocean-based solutions in MPAs: coastal wetlands protection, coastal wetlands restoration, macroalgae protection, macroalgae restoration, seafloor protection, and seaweed farming. The carbon removal and avoided emissions achieved by implementing these solutions globally through 2060 were estimated using meta-analysis of existing studies. Applying all six ocean solutions under global implementation scenarios yields total emissions reduction by 2060 of 16.2 ± 1.82 gigatonnes of carbon dioxide equivalent (GtCO_2_-eq) for the plausible scenario and 24.8 ± 2.46 GtCO_2_-eq for the ambitious scenario. That equates to around 2% of the total carbon mitigation needed to meet the Paris Agreement goals of limiting global warming to 2 °C by 2050. Around 70% of this reduction is attributable to carbon removal and 30% to avoided emissions. Enhancing MPAs’ blue carbon potential could be a key contributor to drawing down carbon and could provide many additional benefits to the marine environment and human society, such as rebuilding biodiversity and sustaining food production. However, more regional-scale studies are needed to inform the best strategies for preserving and enhancing carbon removal in ocean sinks.

## Significance

Marine conservation and the establishment of marine protected areas (MPAs) have gained attention as ways to protect and restore ecosystems and rebuild fish populations. They may also play an important role in sequestering carbon and reducing emissions from sources such as habitat degradation. Implementing six strategies for enhancing blue carbon sinks, including establishing MPAs to protect and restore coastal wetlands, macroalgae forests, and seafloor sediments and expand seaweed farming can not only remove significant amounts of carbon and avoid emissions but also bring many more environmental and human-related benefits.

Marine protected areas (MPAs) are highly effective tools for marine conservation (1). Widely acknowledged benefits of MPAs include biodiversity conservation and protection from overfishing and habitat destruction (2, 3). The extent to which MPAs may also address climate change is debated (4–7). Nevertheless, researchers and policymakers recognize that MPAs may have an important role in reducing carbon emissions and increasing carbon sequestration (8–10).

Indisputably, the ocean is an important carbon sink that has already absorbed around one-fourth of all human-generated CO_2_ emissions (11, 12). Many ocean-based solutions to climate change center around the concept of “blue carbon,” referring to the capacity of marine life to draw down CO_2_ from the atmosphere (13–15). Coastal wetlands hold tremendous carbon stocks and have high rates of carbon sequestration (16). While most blue carbon studies to date have focused on coastal wetland ecosystems, subtidal macroalgae forests also may sequester large amounts of carbon. Wild macroalgae take up an estimated 1.5 gigatonnes of carbon (GtC) per year in net primary production and facilitate the export and long-term sequestration of an estimated 0.17 GtC annually (17, 18). Macroalgae’s carbon sink potential magnifies when the sequestration capacity from seaweed farming is added (17).

Marine ecosystems are experiencing degradation and decline worldwide. The estimated annual loss of coastal wetlands globally is around 1 to 3%, resulting from ecosystem conversion, modifications in terrestrial inputs, and climate change (19, 20). Macroalgae forests are also declining globally at an average rate of 1.8% due to climate-related and anthropogenic factors, such as harvesting, eutrophication, and pollution (21, 22). The loss of coastal wetlands and macroalgae forests limits their carbon sequestration potential and makes coastal wetlands the source of greenhouse gas (GHG) emissions (23).

Additionally, recent global estimates show that the ocean is currently storing around 3,117 GtC in the top 1 m of sediment, much of it in Exclusive Economic Zones (EEZs) that are subjected to high exploitation such as bottom-trawling fishery (10, 24). The potential of blue carbon solutions to mitigate climate change, therefore, depends on the fate of the sediments into which the biomass of these carbon sinks is exported and gets stored in the form of organic carbon.

Currently, MPAs cover 6.4% of the global ocean, with 2.7% of the global ocean classified as fully protected (no-take) zones (25). Calls to expand these areas are growing. Protecting 30% of the ocean through MPAs could halt biodiversity loss and deliver substantial carbon drawdown, food security, and other economic benefits and could prevent the further loss of coastal wetland ecosystems (9, 26, 27). Furthermore, there is evidence that MPAs and management actions, such as protecting predators, minimizing kelp harvest, and reducing eutrophication due to runoff, can improve macroalgae forests’ resistance and resilience to the impacts of climate change (6, 28–30). Similar cobenefits could play an important role in expanding regenerative forms of ocean farming, namely seaweed farming. Finally, establishing MPAs that prohibit destructive bottom trawling can help protect seafloor habitats as well as the carbon stored in the sediments.

**Fig. 1.**
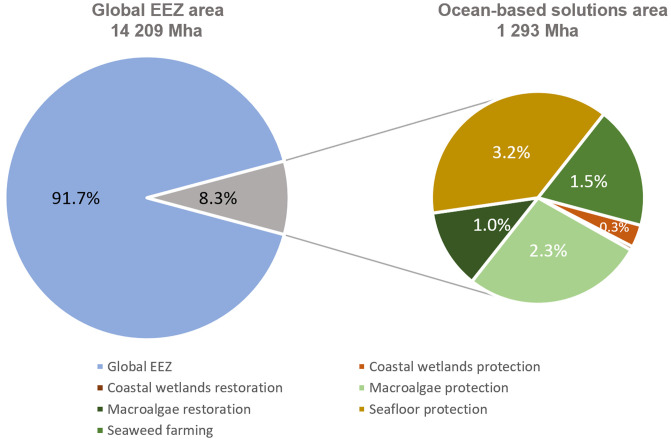
TOA available for each solution presented with the global EEZ area. Note that the TOA represents the maximum adoption potential for solution, not the actual adoption. The actual adoption under plausible and ambitious scenarios is presented in Fig. 3.

**Fig. 2.**
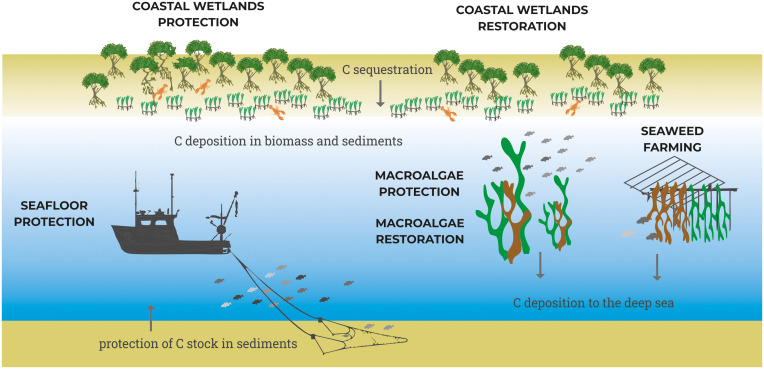
Schematic of ocean-based solutions to climate change. Solutions are oriented around the protection and restoration of blue carbon sinks as well as the expansion of seaweed farming, and all could be implemented within MPAs. Solutions have been identified based on the scientific evidence for long-term carbon removal and avoided emissions, availability of data, and adoption growth in different parts of the world.

Here, we analyze six ocean-based solutions to climate change, as modeled under the Project Drawdown framework ( 31, 32) to determine the potential climate benefit from expanding blue carbon sinks through MPAs. The solutions are coastal wetlands protection, coastal wetlands restoration, macroalgae protection, macroalgae restoration, seafloor protection, and seaweed farming (Fig. 1 and Table 1). Project Drawdown’s models account for carbon removal by sequestration of CO_2_ from the atmosphere into plant biomass and sediment and avoided emissions for a solution relative to a conventional practice. These practices are assumed to require allocation of a specific type of ocean area. The current and potential scenarios are therefore defined in terms of Total Ocean Solution Area (TOA) or the ocean area available for the solution (in millions of hectares; Fig. 2). Impacts on atmospheric greenhouse gases were projected from 2018 to 2060 for three solution scenarios (low adoption, plausible, and ambitious) compared to a reference scenario (*SI Appendix*, Fig. S1).

**Table 1.** List of ocean solutions to climate change modeled under this study with definitions and climate impact mechanism assignments

Solution name	Definition	Climate impact mechanisms
Coastal wetlands protection	The legal protection of carbon-rich mangroves, seagrasses, and salt marshes, leading to reduced degradation rates and the safeguarding of carbon sinks. This solution secures otherwise vulnerable coastal wetlands whose destruction would be a source of greenhouse gasses.	Carbon avoided emissions and carbon removal
Coastal wetlands restoration	Any process that aims to return a coastal wetlands ecosystem to a preexisting condition, whether or not it was pristine. This includes both natural restoration and human-led recovery of carbon-rich mangroves, seagrasses, and salt marshes. This solution recovers coastal wetlands ecosystems’ capacity as carbon sinks.	Carbon removal
Macroalgae protection	The legal protection of wild macroalgae forest ecosystems to secure and enhance long-term sequestration of the carbon exported to the deep sea and/or stored in the ocean shelves.	Carbon removal
Macroalgae restoration	Processes or programs designed to return wild macroalgae forest ecosystems to a previous state from a degraded condition in order to enhance long-term sequestration of the carbon exported to the deep sea and/or stored in the ocean shelves.	Carbon removal
Seafloor protection	The legal protection of high in organic carbon seafloor sediments from disturbance by bottom-trawling fishery, leading to reduced carbon emissions from disturbed sediments.	Carbon avoided emissions
Seaweed farming	The culturing, cultivation, and harvesting of different macroalgae species in the ocean area with the purpose of accounting for the long-term sequestration of the carbon naturally exported to the deep sea and/or stored in the ocean shelves (a significant proportion of carbon fixed by macroalgae is photosynthetically released into the water, of which portion gets distributed below the ocean mixing layer).	Carbon removal

We provide evidence that enhanced ocean protection and restoration focused on blue carbon habitats can achieve significant carbon removal by enhancing carbon sequestration and avoided carbon emissions. This study represents an advancement from previous works because it develops and compares alternative scenarios for a suite of ocean solutions to climate change, including seafloor protection and macroalgae protection and restoration, which have not been included in previous assessments (8). Some of the proposed solutions, such as restoration and protection of coastal wetlands, benefit from extensive past research related to their potential for carbon storage, while others, such as macroalgae-related solutions, should be treated with higher uncertainty.

## Results

The avoided carbon emissions and carbon removal achieved by enhancing the adoption of ocean protection, habitat restoration, and seaweed farming globally over the coming decades were estimated using meta-analysis of existing studies (Table 2). The collected literature includes journal articles, working papers, and professional reports. In total, 169 data points from 114 sources were used (Table 2). Collected studies contain sufficient information to be included in the statistical meta-analysis and are listed in *SI Appendix,* section S5 with data quality assessment.

**Table 2.** Climate impact variables of all solutions obtained from meta-analysis with the number of data points and sources used listed

Solution name	Carbon removal (t ha^−1^ yr^−1^)	Carbon avoided emissions (t ha^−1^ yr^−1^)	Carbon storage in protected ocean area (tC ha^−1^)	Number of data points	Number of sources
Coastal wetlands protection	Mangroves 1.91 ± 0.55	Mangroves 32.75 ± 27.35	Mangroves 585.77 ± 375.51	9, 19*, 9^†^	7, 11*, 4^†^
	Seagrasses 1.19 ± 0.5	Seagrasses 3.81 ± 2.31	Seagrasses 234.60 ± 94.90	2, 9*, 2^†^	1, 6*, 1^†^
	Salt marshes 1.92 ± 0.97	Salt marshes 14.28 ± 15.60	Salt marshes 355.07 ± 301.11	27, 8*, 4^†^	11, 5*, 4^†^
Coastal wetlands restoration	Mangroves 6.58 ± 2.58	—	—	17	12
	Seagrasses 1.00 ± 1.32	—	—	12	4
	Salt marshes 0.93 ± 0.50	—	—	9	6
Macroalgae protection	0.97 ± 0.68	—	—	14	6^‡^
Macroalgae restoration	0.97 ± 0.68	—	—	14	6^‡^
Seafloor protection	—	13.83 ± 6.36	14,745.67 ± 6,597.6	111	55^‡^
Seaweed farming	3.21 ± 2.1	—	—	44	16^‡^

The values are mean with a range defined as one SD above and below the mean of entered values. Carbon removal and carbon avoided emissions are annual estimates. For a detailed description behind each variable, see *SI Appendix*, *S5*.

*Values represent the number of data points and references used in estimating emissions reduction for coastal wetlands protection.

^†^Values represent the number of data points and references used in estimating carbon storage in protected ocean area for coastal wetlands protection.

^‡^Climate impact variables are results of Project Drawdown’s calculations specified in *SI Appendix*, *S1* and not raw data points taken from the published sources.

**Fig. 3.**
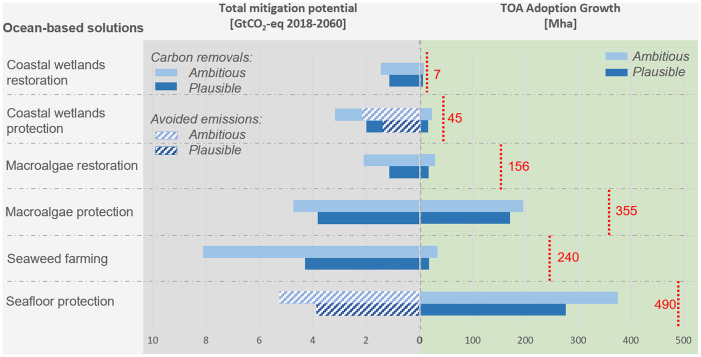
The total mitigation potential (GtCO_2_-eq 2018 to 2060) and TOA adoption growth (Mha) for all six ocean-based solutions under the plausible and ambitious scenarios. Maximum TOA (Mha) assigned to each solution is listed on the right-side of the graph with a red dashed vertical line. Note that results for the low adoption scenario are not included in this figure, as they are much lower and would not be visually compelling. Low adoption results are presented in Table 3 and in the *Results*.

Three scenarios of MPA expansion were evaluated. The low adoption scenario, defined by current trends for MPA establishment, resulted in 10.3 million hectares (Mha) of additional adoption of coastal wetlands protection, 40.5 Mha of macroalgae protection, and 62.9 Mha of seafloor protection (Fig. 3). Under the plausible scenario, the solutions are adopted at an ambitious but realistically vigorous rate, derived from the average of a few global projections; and under the ambitious scenario, the adoption of solutions is increased to the high range of the projections, thus representing an advancement of the plausible scenario (*SI Appendix*, Table S2). The plausible scenario resulted in 15.5 Mha of additional adoption of coastal wetlands protection, 171.1 Mha of macroalgae protection, and 276.6 Mha of seafloor protection (Fig. 3). The ambitious scenario resulted in 22.5 Mha of additional adoption of coastal wetlands protection, 195.4 Mha of macroalgae protection, and 374.5 Mha of seafloor protection (Fig. 3).

The other solutions considered (the restoration solutions and seaweed farming) have much less potential area of implementation. Under the low adoption scenario, coastal wetlands restoration grew by 4.6 Mha, macroalgae restoration by 3.9 Mha, and seaweed farming by 0.7 Mha. Under the plausible scenario, coastal wetlands restoration grew by 6.1 Mha, macroalgae restoration by 16.3 Mha, and seaweed farming by 13.4 Mha. Under the ambitious scenario, coastal wetlands restoration expanded by 7.2 Mha, macroalgae restoration by 28.9 Mha, and seaweed farming by 25 Mha (Fig. 3).

The total area dedicated to all ocean solutions amounted to 122.9 Mha under the low adoption scenario, 502.9 Mha under the plausible scenario, and 661.2 Mha under the ambitious scenario. These represent 10%, 39%, and 51% of the TOA evaluated, respectively.

**Fig. 4.**
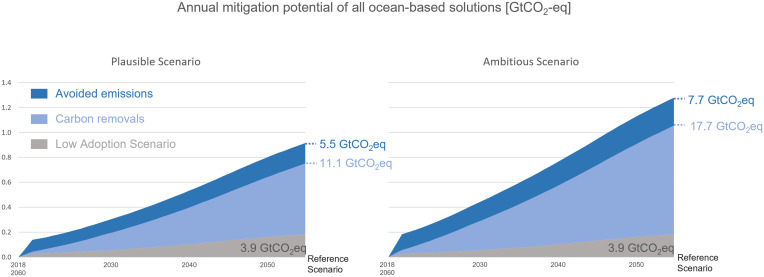
Annual mitigation potential from carbon removal and avoided emissions achieved by applying all six ocean solutions under the plausible and ambitious scenarios compared to the low adoption scenario (carbon avoided emissions and carbon removal impact combined). Total avoided carbon emissions and carbon removal between 2018 and 2060 are presented and indicated with dashed lines.

Applying all six ocean solutions achieved a total carbon reduction for 2018 to 2060 of 16.2 ± 1.82 gigatonnes of carbon dioxide equivalent (GtCO_2_-eq) for the plausible scenario, with 67% of the emissions reduction coming from carbon removal and 33% from avoided emissions. In the ambitious scenario, 24.8 ± 2.46 GtCO_2_-eq were reduced, of which 70% represents carbon removal and 30% represents avoided emissions (Fig. 4). The low adoption scenario resulted in total carbon reduction of 3.9 ± 0.41 GtCO_2_-eq (Fig. 4). The solution with the highest estimated total carbon removal potential is seaweed farming, while the solution with the lowest potential is coastal wetlands restoration (Table 3).

**Table 3.** Solution-specific results of total carbon removal and/or avoided emissions potential achievable between 2018 and 2060 for three scenarios

Solution name	Mitigation potential low adoption GtCO_2_-eq (2018 to 2060)	Mitigation potential plausible GtCO_2_-eq (2018 to 2060)	Mitigation potential ambitious GtCO_2_-eq (2018 to 2060)
	Carbon removal		
Coastal wetlands protection	0.27 ± 0.00	0.57 ± 0.02	0.99 ± 0.02
Coastal wetlands restoration	0.71 ± 0.00	1.1 ± 0.01	1.4 ± 0.01
Macroalgae protection	0.93 ± 0.00	3.8 ± 0.12	4.7 ± 0.14
Macroalgae restoration	0.27 ± 0.00	1.1 ± 0.01	2.1 ± 0.02
Seaweed farming	0.06 ± 0.00	4.3 ± 0.04	8.1 ± 0.07
	Carbon avoided emission		
Coastal wetlands protection	0.67 ± 0.00	1.42 ± 0.04	2.71 ± 0.05
Seafloor protection	1.04 ± 0.40	3.9 ± 1.81	5.2 ± 2.46

The costs of the protection and restoration solutions are assumed to be borne at the government level, and it is assumed that seaweed farming requires private investment. The average first costs for seaweed farming calculated within this study amount to US$9,705 per hectare established per year (with a range of US$3,387 to US$22,798), and the average annual operating costs are US$11,585 per hectare per year (with a range of US$1,847 to US$25,019). The net profit margin amounts to US$12,892 per hectare per year (with a range of US$1,450 to US$25,640).

## Discussion

Highlighting the climate benefits obtained by protecting blue carbon ecosystems may help strengthen the case for establishing MPAs (32). By implementing ocean protections targeted to blue carbon sinks, we could achieve from 10.9 ± 0.13 to 17.4 ± 0.16 GtCO_2_-eq of carbon removal globally in around 40 y depending on the adoption scenario. In the same timeframe, we could also avoid from 5.3 ± 1.8 to 7.4 ± 2.5 GtCO_2_-eq emissions depending on the adoption scenario. Taken together, the potential climate impact stemming from these blue carbon solutions is equivalent to the total emissions of the country of Australia over 40 y (based on 2019 emissions level). The estimated carbon removal potentials differ in terms of uncertainty because carbon removal processes for coastal wetlands protection and restoration have been studied for many years, while data regarding long-term carbon sequestration in macroalgae forests are more limited. Subtracting the less certain solutions (macroalgae protection/restoration and seaweed farming) from the cumulative results reduces the total carbon removal impact by 40% in around 40 y.

Taking advantage of MPAs’ potential as a climate solution will require strategic design and restoration programs focused on preserving and restoring coastal wetlands, macroalgae forests, and seafloor disturbed by bottom-trawling habitats. The estimated climate impact can be obtained by adopting 17 to 39% of TOA as MPAs by 2030 and reaching higher protection targets by 2060 as well as by expanding the more regenerative forms of seaweed farming (such as farming seaweed or seaweed integrated with other species) from 0.19 to at least 17.5 Mha globally by 2060 (Fig. 3). Regenerative seaweed farming does not require any nutrient inputs in the production process so relies on nutrients naturally occurring in the ocean and might locally increase biodiversity by providing complex habitat (33). The annual carbon reduction (including emissions avoided and carbon removal) coming from the application of all six solutions reaches 0.2 to 0.4 GtCO_2_-eq in 2030, 0.6 to 0.9 GtCO_2_-eq in 2050, and 0.7 to 1.1 GtCO_2_-eq in 2060 (plausible and ambitious scenarios, respectively). This figure is close to the low end of annual emissions reductions estimated by Hoegh-Guldberg et al. for blue carbon ecosystems (which only include coastal wetlands protection and restoration and increased seaweed production), which were 0.32 to 0.89 GtCO_2_-eq in 2030 and 0.5 to 1.38 GtCO_2_-eq in 2050 (8). This work builds on a meta-analysis of existing data sources, with adoption cases based on current trends or targets, a somewhat conservative approach that can lead to discrepancies from other studies, such as our ambitious scenario falling behind the Hoegh-Guldberg et al. adoption estimates (8). Taking a conservative approach is appropriate for building realistic and vigorous prognostications, but it also suggests that ocean protection likely offers even greater potential for climate impact than we project.

Reaching plausible or ambitious scenario targets is necessary to achieve significant climate benefits. Without ambitious, sustainable ocean management, the reduction potential of blue carbon habitats will weaken, decreasing their climate mitigation as presented by the low adoption scenario (Fig. 3). The urgency of the climate crisis requires that every effort should be taken this decade to achieve the Paris Agreement targets and limit global warming to 2 °C. The estimated climate benefit of the six blue carbon solutions assessed could provide around 2% of the total carbon mitigation needed to meet the Paris Agreement goals of limiting global warming to 2 °C by 2050, indicating the importance of ocean carbon sinks in solving the climate crisis as urgently, safely, and equitably as possible (34). The potential for integrating ocean-based solutions in global warming mitigation efforts of different countries is growing, as many countries have called to include ocean components in their Nationally Determined Contribution (35). Targeting blue carbon sinks might be especially beneficial for small island states seeking to meet their net-zero targets (36). These protection, restoration, and ocean sustainability practices should not end when the Paris Agreement concludes in 2050 either, as they will continue to provide additional carbon removal and avoided emissions benefits after that, as presented in Fig. 4.

Adopting all six ocean solutions for climate change requires protecting and restoring blue carbon habitats as well as expanding seaweed farming. Combined, these solutions reach 4% and 5% of the global ocean area covered by EEZs under the plausible and ambitious scenarios, respectively (Fig. 3 and Fig. 2). From a global perspective, the area required for adoption is not very large; current global conservation targets call for strictly protecting 30% of countries’ EEZs by 2030 (27, 37). Even though blue carbon habitats are known for their important role in ecosystems and carbon storage, and their area is small compared with that of EEZs, their current level of protection is low. Implementing MPAs might be challenging in some places, such as near coasts populated by those who rely on fishing for subsistence or lack the funding and capacity to do so (7). Including blue carbon conservation goals within MPAs has the potential to add value to the conservation approach, especially if those habitats are included in national GHG accounting.

This is already occurring for coastal wetlands (38). Coastal wetland blue carbon ecosystems are included as a climate strategy under the United Nations Framework Convention on Climate Change and as a biodiversity strategy under the Convention on Biological Diversity and the Ramsar Convention, so policy mechanisms for their protection and restoration are already in place. However, more effort is needed to recognize the full potential impact of blue carbon sinks in all relevant conventions and agreements. It is important to recognize this as we enter the United Nations Ocean Decade and engage different stakeholders in delivering sustainable ocean solutions. Blue carbon solutions can not only mitigate climate change but also contribute to climate adaptation by protecting coasts from erosion and extreme weather events (39) and lessening ocean acidification. Moreover, reaching the adoption targets discussed above would bring many additional benefits to the marine environment and human society in line with the Sustainable Development Goals (40), such as rebuilding biodiversity and habitat complexity (41) and sustaining stable food production (42).

### Source-Specific Climate Benefits.

It has already been noted that the role of offshore and deep ocean benthic communities in climate warming mitigation is underemphasized (43). The current analysis provides evidence that expanding protection of the seafloor from bottom trawling avoids the release of a significant amount of carbon stored in the sediments. A recent global analysis produced a conservation planning framework to prioritize highly protected MPAs in places that would result in multiple benefits, including biodiversity, food provision, and carbon storage in sediments (10). It showed that eliminating 90% of the present risk of carbon disturbance due to bottom trawling would require protecting 3.6% of the ocean in strategically implemented MPAs (mostly within EEZs) (10). The current analysis suggests that out of the 490 Mha of seafloor currently disturbed by bottom trawling, the protection of 276.6 to 374.5 Mha is realistically achievable via no-take MPAs or bottom trawling bans and results in significant climate impact across all six ocean solutions.

MPAs also have a high potential to secure carbon and enhance carbon sequestration within coastal wetland habitats and macroalgae forests. This analysis suggests that coastal wetland protection and restoration make a moderate contribution to climate change mitigation, together removing between 1.7 ± 0.01 and 2.4 ± 0.02 GtCO_2_-eq and avoiding between 1.4 ± 0.04 and 2.2 ± 0.05 GtCO_2_-eq in around 40 y, which is impressive given the relatively tiny area covered by coastal wetlands (Fig. 3 and Table 3). Wild macroalgae forests cover significantly larger areas globally but have estimated climate benefits similar to that of coastal wetlands, amounting to carbon removal of 4.9 ± 0.11 to 6.8 ± 0.14 GtCO_2_-eq in around 40 y (Fig. 3). Many anthropogenic stressors, such as coastal conversion to urban uses, eutrophication, algae harvesting, and fishing and climate-warming stressors can be addressed by local resource managers and may be ameliorated via protection in MPAs (29, 30, 44–46). However, even with the best efforts of local resource managers to conserve these ecosystems, some continued decline due to climate change is likely inevitable (46, 47). The climate change effects that have negatively affected coastal wetlands and macroalgae include increased water temperature and heat wave events (48, 49) as well as sea level rise (50). The loss of coastal wetlands and macroalgae forests that may occur despite their protection in MPAs underscores the importance of incorporating active habitat restoration into MPA design, an essential part of management planning that could provide an estimated removal of between 2.3 ± 0.01 and 3.5 ± 0.02 GtCO_2_-eq from 2018 to 2060.

Highly protected MPAs by definition do not allow for commercial activities (51). However, integrating regenerative seaweed farming within MPAs may bring significant climate and socioeconomic benefits for coastal populations (52). Seaweed farming is a considerable climate solution, with an estimated impact of 4.3 ± 0.04 to 8.1 ± 0.07 GtCO_2_-eq removed in around 40 y (Fig. 3 and Table 3). Most carbon absorbed by seaweeds is consumed or recycled in shallow-water ecosystems, and only that portion that is exported and sequestered into deep-sea sediments or below the mixed layer can be considered sequestered long term. Present studies only account for the long-term sequestration of the carbon naturally exported to the deep sea and/or stored in the ocean shelves (Table 2 and *SI Appendix*, section S5). In 2005, global macroalgae production was estimated at 14.7 million metric tons; by 2016, global yields had more than doubled, reaching 31.2 million metric tons and comprising 27% of total marine aquaculture production (53). The potential for seaweed farming expansion is similarly high, with the top-producing nations as well as new nations focusing on ensuring the sector’s long-term sustainability (42, 54). Many coalitions and nongovernmental organizations (NGOs) are providing guidance and support to farmers to ensure sustainable and innovative seaweed production. Moreover, seaweed biomass is used in an increasing number of applications, including animal feed (55), bioplastics, and biofuel (56). This is unlocking the potential for market growth and is creating opportunities to reduce GHG emissions from other sectors, for example, by adding seaweed to livestock feed to reduce methane emissions from cattle (57).

### Financial Implications of Ocean Solutions.

Implementing MPAs requires funding and safeguarding mechanisms to ensure the longevity of protection and restoration projects. Funds can come from nonmarket mechanisms, such as convention funds and national or multilateral development funds, and from market mechanisms, such as regulated or voluntary carbon markets. Coastal wetlands are beginning to be included in carbon credit schemes. The potential for marketing blue carbon ecosystems is high; the global blue carbon wealth (defined as carbon sequestration and storage potential) is estimated to amount to over US$190 billion per year (58).

For the scope of the current analysis, the costs of protection and restoration projects are assumed to be borne at a government and NGO level (59). Governments often have the resources necessary for project implementation, while NGOs and other trusted institutions have social influence that can increase support from local communities (60). Costs would range widely depending on the size of a project, methods applied, and location; for example, the average cost of coastal wetland restoration projects in developing countries was estimated to be US$80,000 to US$1.6 million per hectare of restored area, with seagrass projects being the most expensive and mangrove projects being the least expensive (59). Similarly, a wide range is observed for macroalgae forest restoration projects that run for at least a few years, with budgets spanning from US$5 million for small, short-term projects to US$267 million for a large project that aims to restore 54,000 ha in Korea within 15 y (60). Seaweed farming is the only solution modeled in this study in which it is assumed that private funding is required. Currently, data on the costs of seaweed farm installation and operation are limited. A review of the financials of eight farming systems from developing countries concluded that the costs of seaweed farming largely depend on scale (family size versus industrial) and location (61); the present study confirms the high range of seaweed farm installation and operation costs (*Results*). Similarly, profit from these farms will vary, as it largely depends on the application of harvested seaweed biomass. To further advance the application of biomass, a growing number of NGOs and coalitions are providing support and connecting seaweed producers with suppliers to ensure that demand is met with supply (42).

Closing ocean areas for certain activities such as bottom trawling could have implications for the fisheries sector. Considering the importance of fisheries employment, MPAs should be designed in a way that mitigates adverse economic impacts. In particular, area closures and resulting job loss might disproportionately affect low-income or otherwise marginalized fishing communities. An Organization for Economic Cooperation and Development bioeconomic model found that simply redirecting fisheries subsidies from capacity-enhancing subsidies to other types (e.g., from fuel subsidies to direct income payments to fishermen) could help achieve fishing effort reduction and stock-rebuilding goals while increasing the total income of fishers, especially for small-scale fisheries (62). Many bottom-trawling fisheries rely on capacity-enhancing subsidies, without which their operations would not be profitable (63). Therefore, redirecting harmful fishery subsidies into fishers’ direct payments or training for alternative employment opportunities could be more beneficial (64).

### Limitations and Barriers to Achieving Adoption.

Resolving conflicting biological and socioeconomic objectives remains one of the major challenges of ocean management (65). Scaling up ocean protection efforts to achieve regional and global impact requires encouragement and support from governments and development agencies using appropriate legal, financial, and social incentives. Such support should be considered part of national and international commitments regarding climate change adaptation and mitigation.

Potential shortcomings of MPAs or bottom-trawling closures include insufficient staff, equipment, and funding, inadequate consultation with local communities, and concerns about managing displaced fishing effort (7). Therefore, MPA design and management should include local communities and be integrated with other management tools, such as territorial user rights (66). Excluding the most damaging activities (e.g., industrial fishing) within MPAs and favoring small-scale fisheries could benefit local communities not only in the potential increase in catch and profits from tourism but also in potential adaptation to climate change (7). Although governments are likely to bear the brunt of the MPA implementation economic impact, the ecological and climate benefits might outweigh the costs if climate change mitigation and adaptation benefits are included.

Limited scientific data might delay the creation of mechanisms to incorporate ocean-based solutions into existing climate mitigation programs. A lack of representative data and well-established measurement methodologies restricts the scientific understanding of global carbon fluxes from ocean ecosystems (67). Multiple, inconsistent approaches for defining mangrove forests and measuring carbon in coastal wetlands also present challenges for determining carbon sinks and fluxes. Data on long-term carbon sequestration in macroalgae forests and the fate of carbon remineralized due to bottom trawling are even more limited, so estimates of carbon sequestration and emissions reduction potential for these solutions are less certain than those for coastal wetlands (10, 17, 68). Moreover, the current study uses global estimates of carbon sequestration or emission, which vary widely depending on the local environment, for example, by hydrodynamic regimes (69, 70), climate zone, and season (71, 72); species, for example, Posidonia versus Zostera species (67); and plant development, for example, density of seagrass meadows (73) and mangrove height and biomass (71). Still, the carbon removal potential of macroalgae-related solutions and emission reduction potential of seafloor protection might be high (Fig. 3 and Table 3), and more effort should be taken to increase research and development of these solutions. Specific areas of research that would improve current results include local measurements of carbon sequestration and emissions under different environmental and ecological settings.

The current modeling framework provides a tool for estimating the climate change mitigation potential of blue carbon habitats globally and at different scales. It also highlights the potential carbon solutions that countries and communities can explore as part of their MPA strategy and coastal management. However, more regional studies are needed to support the successful implementation of these solutions to preserve and enhance carbon sequestration in ocean sinks.

## Materials and Methods

We used four criteria to identify potential ocean-based solutions for evaluation: 1) there is scientific support for their ability to avoid carbon emissions and provide long-term carbon removal (deposited in the sediment from 100 to 1,000 y and/or exported below the ocean mixing layer), 2) there is a business case for the solution and/or adoption growth in different parts of the world, 3) data exist to enable modeling of the solution, and 4) the solution’s benefits outweigh negative externalities. Several assumptions were made in applying the Project Drawdown methodology (31), including 1) the infrastructure required to scale each solution globally is already in place and is embedded in the cost to the agent (e.g., the individual/household, community, city, or utility); 2) policies required to enable, augment, or regulate solutions at the local, national, and international level are already in place; and 3) many solutions may become outdated, significantly improved, or supplanted by new technologies or practices within the period under analysis, but that is not factored in due to the absence of reliable data.

The basic unit of analysis is the TOA, defined as the area of ocean suitable for adoption by solutions in Mha. TOA is specific to each solution and represents the upper limit of possible adoption. The TOA projected for all solutions, based on a literature review (*SI Appendix*, section S2 and Table S1), was 1,297 Mha or 8.3% of the 14,209 Mha global EEZ area (74) (Fig. 2).

The percent of the TOA that is protected, restored, or designated to seaweed farming in 2018 was chosen as current adoption based on the availability of reliable data. Increased adoption of TOA was projected until 2060 for each solution. Three scenarios were developed with different levels of increased adoption and compared with a reference scenario in which those adoptions remain fixed at the 2018 level. The TOA and adoption cases were derived by reviewing literature from authoritative sources, including peer-reviewed journals, public sector and multilateral agencies, and other nongovernmental organizations (*SI Appendix*, sections S2 and S3 and Tables S1 and S2). The three scenarios were defined as follows: low adoption scenario (ocean protection, restoration, and seaweed farming are adopted at historical growth rates), plausible scenario (ocean protection, restoration, and seaweed farming are adopted at an ambitious but realistically vigorous rate, derived from the average of a few global projections), and ambitious scenario (adoption of ocean protection, restoration, and seaweed farming is increased to the high range of the projections, thus representing an advancement of the plausible scenario; *SI Appendix*, Table S2).

The climate impact achieved by increasing the adoption of ocean sinks protection, restoration, and seaweed farming was projected by calculating the carbon sequestration (carbon removal) and/or avoided emissions of each solution separately. Data for these variables were obtained via meta-analysis of various sources, with the total number of estimates and sources listed in Table 1 and detailed descriptions of per solution calculations presented in *SI Appendix*, section S5. The total removal of CO_2_ is captured in the sequestration rate of the solution. These sequestration rates are used to estimate the amount of sequestration that happens annually and are assumed to be constant over time. The sequestration is defined as

$$\sum_{y=y1}^{y_{\text{max}}}Sy^{{\text{CO}}_{2}}=\sum_{y=y1}^{y_{\text{max}}}\frac{3.666\cdot q\;\cdot (\mathrm{NAOUy}-Ty)\cdot (1-\delta )}{1,000},$$

where [*y*1, *y*_max_] is the range of years of the analysis (2020 to 2050), *Sy*^CO2^ is the total CO_2_ sequestered for year *y* in GtCO_2_-eq, *q* is the sequestration rate for solution over conventional practice in tonnes of carbon per hectare (tC ha^−1^ yr^−1^), *NAOUy* is the net annual ocean units (the solution adoption in the solution scenario minus the solution adoption in the reference scenario in year *y* in Mha), *Ty* is the total ocean that is harvested in year *y* for its produce (applicable only for seaweed farming solution) in Mha, and *δ* is the disturbance rate for solution in percent.

Total carbon avoided emissions is linear to the number of ocean units adopted, with emissions efficiency of the solution a fixed value. It is a linear sum of the avoided emissions from direct source (*SI Appendix*, section S5)

$$\sum_{y=y1}^{y_{\text{max}}}R_{y}{}^{{\text{CO}}_{2}\text{-}\text{eq}}=\sum_{y=y1}^{y_{\text{max}}}\frac{\mathrm{NAOUy}\;\cdot \;\left\{ER\right\}\;\cdot (1-\delta )}{1,000}\;,$$

where *Ry*^CO2-eq^ is the total reduction in emissions of gas in year *y* in GtCO_2_-eq, and *ER* is the reduction in other direct emissions (beyond grid and fuel emissions) when the solution is used instead of the conventional practice in tonnes of carbon dioxide equivalent per hectare (t CO_2_-eq ha^−1^).

To account for uncertainty in analyses, error propagation of final estimates has been applied (*SI Appendix*, section S6). The total additional carbon removal or carbon avoided emissions was estimated by comparing the three scenarios to the reference scenario (*SI Appendix*, section S6).

## Supplementary Material

Supplementary File

## Data Availability

All data included in the article are coming from published sources and are cited in the main text and in the *SI Appendix*.
