# Supplementary material for: Climate benefits from establishing marine protected areas targeted at blue carbon solutions
Source: Proc Natl Acad Sci U S A. 2022 Jun 2;119(23):e2121705119. doi: 10.1073/pnas.2121705119 (PMC9191663; doi:10.1073/pnas.2121705119)
Supplement: Supplementary File [file pnas.2121705119.sapp.pdf]

PNAS
www.pnas.org

Supplementary Information for

Climate benefits from establishing Marine Protected Areas targeted at blue carbon solutions.

Emilia Jankowska, Robin Pelc, Jimena Alvarez, Mamta Mehra, Chad J. Frischmann

Email: chad.drawdown@gmail.com

This PDF file includes:

Supplementary text S1 to S3
Figure S1
Tables S1 to S3
SI References

Supplementary Information Text

S1. Modelling framework

Project Drawdown is a research-based organization with a mission is to help the world reach "drawdown" -- the point in the future when levels of greenhouse gases in the atmosphere stop climbing and start to steadily decline, thereby stopping catastrophic climate change--as quickly, safely, and equitably as possible. Project Drawdown has developed models to examine the emissions and financial impact of ambitious but plausible adoption of existing climate solutions. The goal is to see what it would take to get to drawdown with these technologies and practices that are viable, scaling, and financially feasible.

Project Drawdown's models accounts for carbon removal by sequestration of carbon dioxide from the atmosphere into plant biomass and soil as well as reduction of emissions for a solution relative to conventional practice. These practices are assumed to use a specific type of ocean, which may be shared by several solutions (e.g. coastal zone). The actual and maximum possible adoptions are therefore defined in terms of ocean area. The adoption of conventional practices and solutions was projected for each of several adoption cases from 2018 to 2060, and the comparison of three solution scenarios (*Low Adoption, Plausible*, and *Ambitious*) to a *Reference* scenario constituted the results (Figure S1). The results were obtained by following a step-by-step approach presented in Figure S1 and described below.

S2. Total Ocean Solution Area

The starting point of the analysis was setting the Total Ocean Solution Area (TOA), defined as the area of ocean suitable for adoption over time globally by solutions in million hectares (Mha). TOA is a specific ocean area for each solution and represents the upper limit of possible adoption (Fig. S1). This means that even though the TOA for a solution could be high (e.g., for seaweed farming [Fig. 1, Fig. 3]), the actual adoption -- which depends on historical growth rates or national commitments -- can remain low (Fig 3, Fig. S1). The customized TOA was projected for all solutions by obtaining the data from the literature review (Table S1). The underlying assumption for TOA projection is that the ocean area designated for a given solution had no previous use, so no ocean-use transformations are required.

S3. Adoption cases

The next step of the analysis was building adoption cases for each solution, which represent a range of possible adoption (based on historical growth or national commitments) applied to the TOA. The specific features of the adoption cases were developed from review and analysis of current literature (Table S2). Defining an adoption case requires balancing projections of advocates for greater ocean protection (who might provide projections that are implausibly optimistic) with the projections of others, which may be more feasible but perhaps not optimistic enough. The core assumption inherent in this approach is that all policy and financial levers, infrastructure development, and consumer behavior required to reach the specified levels of adoption will be implemented. For all protection solutions, adoption cases were developed by applying marine protected areas (MPAs) or bottom-trawling bans at a global level from 2018 to 2060 using the best available data and forecast information (Table S2). For the two restoration solutions, adoption cases were developed using the effectiveness of historical restoration projects (Table S2). For the seaweed farming solution, adoption cases were developed using a possible increase in the farming areas globally (Table S2). Adoption cases were applied to the TOA specific for each solution, which is defined in Table S1.

S4. Scenarios and climate impact results

After developing the TOA and adoption cases for each solution, four scenarios were run:

- *Reference* scenario, in which adoption was fixed at the percent adoption in the specified current year (2018). The percent of solution adoption was kept constant throughout the study period (until 2060). This serves as the baseline of comparison for the *Low Adoption, Plausible,* and *Ambitious* scenarios (Fig. S1).

- *Low Adoption* scenario, in which solutions are adopted at the historical growth rate or lowest adoption case. This scenario represents the future state of protection and restoration with the current level of ambition.

- *Plausible* scenario, in which solutions are adopted at a realistically vigorous rate over the time period under investigation. A complete analysis was performed for the *Plausible* scenario using a rate of TOA adoption that is the mean of all adoption cases for a specific solution (listed in Table S2).

- *Ambitious* scenario, in which the adoption of solutions is accelerated beyond the *Plausible* scenario. The same analysis was performed for the *Ambitious* scenario as for the *Plausible* scenario; however, the rate of TOA adoption used was the high estimate of all adoption cases for a specific solution (listed in Table S2).

S5. Climate variables

The climate impact achieved by increasing the adoption of ocean sinks protection, restoration, and seaweed farming was projected by calculating the carbon removal or avoided emissions of each solution. Data for these variables were obtained from meta-analysis of various sources, as documented in Table 1 and below. In all cases the sources were obtained via Google Scholar search. All sources were included in the analysis and tested for quality: peer-reviewed sources were prioritized (other sources were used only when the data for a variable lacked peer-reviewed sources); the most recent sources were prioritized over older sources (particularly those published before the year 2000, as the measurements techniques changed over time); and the final list of sources was vetted by experts from the relevant fields. All obtained data points were used to derive the global average for each climate impact variable. Only the coastal wetlands solutions consist of published data for regional sequestration rates; the other solutions lack this kind of granularity. Therefore, to unify the approach, a global average (with standard deviation) was applied to climate impact calculations for all solutions. To provide a level of sensitivity analysis, carbon removal and avoided emissions estimates for three scenarios were developed based on a range around the mean. In the model, the low-to-high range is a user-defined number of standard deviations above and below the mean of entered values (in this case one standard deviation), which allows for the identification and elimination of outlier data points. The approach and calculations to estimate the main climate impact variables differed for each solution and are described below, with the final carbon removal [t ha^{-1} yr^{-1}] or carbon avoided emissions [t ha^{-1} yr^{-1}] estimates presented in Table 1.

Coastal wetlands protection

Many of the obtained data points for coastal wetlands are results of global meta-analyses using different methods and primary sources. Those data points are usually based on hundreds of primary sources, some of which are reused in different studies. The reuse of primary sources makes it extremely difficult to determine the number of unique primary sources contributing to our estimate. This difficulty applies to other climate variables as well. In the current analysis, the number of values averaged together for input into the Drawdown model are presented (Table 2). These “numbers of data points” are likely one to two orders of magnitude smaller than the number of primary observations that they are derived from. Sequestration rates for mangroves are based on a meta-analysis of 9 data points from 7 sources (14, 72, 76–80). Eight data points from 6 sources were excluded from the average calculations, as they were already incorporated in or updated by

more recent global studies (14, 76–80). Emissions from degraded or deforested mangroves (which are computed as a climate benefit given that protection would avoid these emissions reaching the atmosphere) are based on a meta-analysis of 19 data points from 11 sources (14, 81–90). Seven data points from 3 sources were excluded from the average calculations, as they were already incorporated in or updated by more recent and peer-reviewed global studies (23, 82, 91). Mangrove carbon storage is based on 9 data points from 4 sources (80, 81, 92, 93). Two data points from 2 sources were excluded from the average calculations, as they were already incorporated in or updated by more recent global studies (93, 94). Salt marsh sequestration rate is based on 27 data points from 11 sources (14, 16, 72, 76, 95–101). Emission from degraded salt marshes is based on 8 data points from 5 sources (23, 68, 83, 102, 103). Two data points from 1 source were excluded from the average calculations, as they are not from peer-reviewed studies (104) Salt marsh carbon storage is based on 4 data points from 4 sources (23, 94, 105, 106). Seagrass sequestration is based on 2 data points from 1 source which is a meta-analysis of 155 different sites (107). Six data points from 4 sources were excluded from the average calculations, as they were already incorporated in or updated by more recent global studies (14, 72, 99, 108). Emission from degraded seagrass beds is based on 10 data points from 6 sources (23, 68, 83, 109–111). Seagrass carbon storage is based on 2 data points from 1 source, which is a meta-analysis of 3,640 observations at 946 different locations globally (112).

An initial annual estimate for the climate impact of coastal wetlands protection is 0.45 $GtCO_2$-eq yr^{-1} with an uncertainty range of 0.15 – 1.02 $GtCO_2$-eq yr^{-1} (23). A more recent, study calculates an annual impact of 0.30 $\pm$ 0.16 $GtCO_2$-eq yr^{-1} in 2030 with 0.13, 0.04, and 0.13 $GtCO_2$-eq yr^{-1} for mangroves, salt marshes, and seagrasses, respectively (113). The *Ambitious* scenario for the model presented here results in a maximum annual reduction of about 0.14 $GtCO_2$-eq yr^{-1}, somewhat more conservative than both benchmarks. There are some key differences in the data used by (23) and the data used in this study. Since the analysis of (113) closely parallels the (23) estimates for mangroves and duplicates them for salt marshes and seagrasses, we do not discuss it separately here. First, (23) used degradation rates based on older sources that can be up to twice as high as the degradation rates used in this study The climate impacts of coastal wetlands protection will be greater if the degradation rates are higher. Secondly, the older sources feeding into the (23) estimate overestimated the area of mangroves by about 10%. Finally, the (23) calculation used higher values for coastal wetlands' carbon storage, in particular nearly double the values used here for mangroves and salt marshes.

Coastal wetlands restoration

The climate inputs used for this solution correspond to sequestration rates only. The studies used do not measure the emissions of the land before restoration occurred; hence, avoided emissions could not be included. The biosequestration from mangroves includes both aboveground biomass and below-ground carbon. The aboveground biomass sequestration variable is based on 17 data points from 12 sources with an average of 4.0 ± 2.6 t CO_2-eq sequestered ha^{-1} yr^{-1} (114–125). The below-ground carbon sequestration variable is based on 10 data points from 3 sources with an average of 2.6 ± 1.4 t CO_2-eq sequestered ha^{-1} yr^{-1} (118, 126, 127). The saltmarsh sequestration rate is based on 9 data points from 6 sources (127–132). The seagrass sequestration rate is based on 12 data points from 4 sources (109, 111, 127, 133).

(113) calculates an annual impact from coastal wetlands restoration's maximum additional mitigation potential of 0.84 $GtCO_2$-eq yr^{-1} in 2030. The main reason for the considerable difference between (113)'s annual climate mitigation impact and those resulting from our models is the restoration area included in the analysis. The average adoption area by 2030 of our adoption scenarios is 6 Mha, whereas (113)'s is 29 Mha. (113)'s restored area estimates are based on older sources, which included a great range of global area estimates (14, 23). For instance, according to (14, 23), salt marshes' global extent ranged from 2.2 to 40 Mha. Our analysis is based on updated global figures, which are considerably lower: 5.5 Mha (134). Inputting the restored area from our models into (113)'s calculation results in a decrease in mitigation impact from 0.84 to 0.11 $GtCO_2$-eq yr^{-1}. The other difference between (113)'s analysis and ours is that that study includes an

avoidable flux from soil carbon oxidation in degraded coastal wetlands. The studies included in our analysis did not report estimates regarding the emissions from the degraded lands before restoration, thus it was not possible to include this dimension of analysis.

Macroalgae protection and restoration

(17)'s seminal paper was used to estimate the percentage of Net Primary Production (NPP) that results in long-term carbon sequestration in macroalgae forests as well as the global extent of wild macroalgae and NPP values. The long-term sequestration rate is the crucial climate input for the macroalgae solutions, and this rate is much more uncertain than the rate of long-term sequestration in coastal wetlands. Most carbon absorbed by macroalgae is consumed or recycled in shallow-water ecosystems, and only that portion that is exported and sequestered into deep-sea sediments or below the mixed layer can be considered sequestered long-term. (17) synthesized the role of wild macroalgae in marine carbon sequestration and identified four pathways of long-term carbon sequestration: macroalgae buried in the algal bed, dissolved organic carbon (DOC) exported below the mixed layer, particulate organic carbon (POC) buried in the shelves, and POC exported to the deep sea. The combined total long-term carbon sequestration estimate from their study was 11% of total NPP.

To estimate the long-term sequestration, the following equation is used:

$$Sequestration\ rate = NPP \cdot \%\ long - term\ carbon\ sequestration\ from\ NPP$$

Sources for wild macroalgae NPP values were compiled from 14 data points published in five studies (13, 17, 53, 57, 135). Seven data points from 3 sources were excluded from the average calculations, as they were already incorporated in or updated by more recent global studies (17, 57, 135). The average value was 8.5 ± 5.9 tC ha^{-1} yr^{-1}. This average value was multiplied by the percent sequestered, estimated to be 11% by (17) as described above, to calculate carbon sequestration rates.

No study was found that estimated the potential carbon benefits of protecting macroalgae forests. (17) estimated that macroalgae forests sequester up to 173 TgC yr^{-1} (range: 61-268 TgC yr^{-1}). Converted to Gigatons of CO_2-eq, this is equivalent to approximately 0.635 Gt CO_2-eq removed per year. This represents total current macroalgae forest carbon removal, and (17) did not estimate the proportion of this removal that would result from macroalgae forest protection scenarios. Considering that the Drawdown model estimates the climate benefit of protection of some proportion of this macroalgae forest (relative to a *Reference* scenario in which a proportion is degraded) it is expected that about 25-50% of this total removal could be considered a climate benefit of the protection scenarios.

Our model estimates that, relative to a *Reference* scenario, protection of macroalgae forests could annually sequester from 0.16 $GtCO_2$-eq (for the *Plausible scenario*) to 0.33 $GtCO_2$-eq (for the *Ambitious scenario*). Adjusting for the proportion of the total carbon removal potential that might be attributed to macroalgae forest protection (about 25-50%), the comparison of our values with those in (17) is in line with expectations.

Seafloor protection

The emission of carbon from disturbed sediment by bottom-trawling activity is a highly uncertain process that depends on many factors. However, recent advances in the study of global carbon stock in sediment, as well as of bottom-trawling footprint, allow us to approximate the rate of carbon emissions. Also, a recent study demonstrates that trawling disturbances in the sediment layer are associated with a sudden and temporary increase in the mineralization rate of organic matter via the introduction of a new pool of reactive organic matter (136). The main climate input for the current solution, 'Emission of CO_2-eq t ha^{-1} yr^{-1}', thus comes from the following equation:

$$Tons\ of\ CO2\ emissions = C\ storage\ in\ ocean\ seafloor\ [t] \cdot POC\ disturbance\ by\ bottom\ trawling[\%] \cdot penetration\ depth\ of\ trawling\ gear[m] \cdot labile\ organic\ matter\ fraction[\%] \cdot remineralization\ rate\ of\ C\ to\ CO2[\%] \cdot C\ to\ CO2\ conversion\ ratio$$

Carbon storage is taken from the global meta-analysis of over 11,000 data points from 685 sources of carbon content in the sediment cores (24). Those data points were fitted to the bottom-trawling areas of (10) using GIS analysis (137) and EEZ boundaries provided by (138). Then an average of carbon stock was calculated for every region. In total, the average carbon stock within 1 meter of sediment was obtained for 115 regions. The global average carbon stock in 1 meter of sediment is 14,745.7 ± 6,597.6 t ha^{-1}, and the low range has been used for the analysis (8,379.1 t ha^{-1}) as a conservative assumption. The disturbance of the sediment and fraction of organic carbon resuspended also largely depend on the gear type and type of sediment. It is generally assumed that the penetration depth of fishing gear is higher in muddy sediment, but other features may also shape this variable, such as gear type, its dimension, and boat speed (139–141). For example, the sediment penetration of otter trawl has been estimated to be up to 30 centimeters in soft sediment (142), while typical penetration of beam trawls is in the range of 6 to 8 centimeters (143). However, nowadays there are technological measures that can limit the seabed contact of fishing gear, such as electric pulse devices coupled to beam trawls that can be used instead of chains to force targeted species out of sediments (144) or disks attached to otter trawls that raise sweeplines above the seafloor and decrease sediment disturbance (145). In the current analysis, the low estimate of gear penetration depth of 1.5 centimeters (coming from 11 data points from 4 sources) was used as a conservative assumption (10, 143, 146, 147). Three data points from 2 sources were excluded from the analysis, as they do not represent direct measurements but the authors' assumptions (146, 147). To come with the amount of carbon that gets disturbed by the bottom-trawling gear, six studies that measured POC concentration in the upper sediment before and after trawling events were used to derive the estimate of organic carbon resuspension, yielding an average of 6.2 ± 6.0% based on 24 data points from 4 sources (141, 148, 149). Six data points were excluded that presented order-of-magnitude-higher resuspension rates as a conservative assumption. Once resuspended, the organic carbon may be dispersed over large distances along with sedimentsplumes (150), may be deposited back into the sediments and buried down by burrowing organisms, or may be remineralized in aerobic processes (151). The remineralization depends on the quality of organic matter, its origin (allochthonous vs autochthonous), and its lability. Labile organic matter is characterized by low molecular weight, thus is easily assimilated in microbial processes and gets quickly transformed to CO_2 (152). In this analysis, solely the labile fraction of organic matter is assumed to be remineralized following (10). The lower estimate of labile organic matter fraction of 15.4%, derived from 28 data points, has been applied in the equation as a conservative assumption (with average 24.0 ± 8.7 %) (10, 153, 154). Finally, only a small fraction of resuspended and labile organic matter will be remineralized to CO_2. For this analysis, 30.0% is used following the global study of carbon remineralized to aquatic CO_2 due to bottom trawling (10). The standard ratio of the weight of CO_2 relative to that of C was applied (that is, 3.67:1).

Assuming that bottom trawling occurs once a year in a given ocean unit, the key climate input of reduced carbon emissions per ocean unit per year was calculated following the above-mentioned equation. Other key assumptions for this solution include: a) no bottom trawling is happening outside the calculated TOA under both the *Reference* and three adoption scenarios; b) carbon stock is uniformly distributed in the 1 meter sediment column; c) bottom-trawling incidents are happening in the spring/summer season of high productivity, so the likelihood of carbon remineralization is higher than if the disturbance were to happen in the winter season with low productivity and temperatures (this assumption is in line with primary trawling seasons, which in many places occur when production peaks); d) remineralization rate is the same globally despite different temperatures and seasons; e) mixing of the sediments and resuspension increases the amount of time the disturbed carbon is in contact with oxygen.

The final results of seafloor protection climate mitigation have been compared to a recent study estimating sedimentary carbon disturbance due to global bottom trawling (10). Based on their

analysis, the disturbance to the seafloor results in an estimated 1.47 Gt of aqueous CO_2-eq emissions in the first year after trawling, then a decrease in emissions in the following years until they stabilize at 40% (0.58 Gt) after 9 years of continuous trawling (10). When applying (10) estimates to our Project Drawdown Scenarios that assume protection from or banning of bottom trawling resulting in cessation of disturbance-related emissions, the total emission reduction is between 7.5 and 10.2 $GtCO_2$-eq yr^{-1} (2018-2060). This estimate is around 60% higher than Project Drawdown's total emission reduction of between 3.9 and 5.2 $GtCO_2$-eq yr^{-1} (2018-2060). This discrepancy is a result of a conservative approach applied by Project Drawdown that used lower estimates for the fraction of labile organic matter and sedimentary carbon disturbance due to bottom trawling. Project Drawdown has also used simplified analysis due to model constraints and did not factor into our calculations the natural sediment accumulation rates that will add more carbon to the sedimentary pool each year. Finally, Project Drawdown assumes that a fraction of emitted carbon reaches the atmosphere (147), whereas (10) only quantifies emissions of aquatic carbon.

Seaweed farming

(135) and (155) estimated a potential carbon removal from macroalgae farming of 1,500 and 1,110 tCO_2-eq km^{-2} yr^{-1}, respectively. However, these estimates include the carbon in the harvested macroalgae, which some authors denote as "removable carbon" (156). Given that the long-term removal potential of the harvested macroalgae depends on its final use and that, unlike wild macroalgae, macroalgae farms are not attached to the seabed, our analysis only includes DOC exported below the mixed layer and POC either buried in the shelves or exported to the deep sea. In order to estimate the long-term sequestration, the following equation is used:

$$Sequestration\ rate = \frac{yield}{(1 - \%farm\ biomass\ export)} \times farm\ biomass\ export \times carbon\ content\ of\ algae\ biomass \times \%long-term\ sequestration\ from\ exported\ carbon\ in\ farms$$

An input that is significant for climate impact calculations is yield. Yield data shows high variability and depends on farmed species, type of farm, and location. Based on our meta-analysis, the average yield is 24.8 ± 4.4 t dry weight ha^{-1} yr^{-1} based on 46 data points from 15 sources (57, 62, 135, 155–166). Farm biomass export comes from two sources, and the average of two data points amounts to 55.0 ± 7.1%. The carbon content of algae biomass has been collected for different species from 30 data points and 7 sources and averaged out to 28.5 ± 4.2%. Finally, the percentage of long-term carbon sequestration from exported carbon in farms comes from two sources, and the average of two data points was 37.2 ± 18.1% (17, 167). This number has been vetted with experts from the Oceans 2050 team.

The key assumptions for this solution include: a) the adoption area used for macroalgae farming had no previous use, hence there are no climate benefits attained from conventional use of that ocean area; b) the delay in obtaining permits for macroalgae harvesting would be less than a year; c) the solution's expected lifetime -- the time the implementation unit will last before replacement is required -- is 30 years.

The carbon mitigation potential of the solution only includes the exported DOC/POC that goes into the deep sea and/or sediments. This carbon mitigation potential could be an underestimate if the harvested macroalgae were ultimately used as biofuel, feed for livestock, or bioplastics, given the avoided emissions this would achieve in other sectors. The effects of produced macroalgae biomass allocations on climate impact will be calculated further by other Project Drawdown sectors and are not included in the current analysis.

The final results of climate change mitigation impact by seaweed farming have been compared to (8)'s study that developed two scenarios to investigate the mitigation potential of seaweed farming by 2030 and 2050. Scenario 1: seaweed farming increases at 8.3% per year (following the 2000-2017 harvesting evolution by FAO); scenario 2: seaweed farming increases at 14% per year from 2013 onwards. In both scenarios, 100% production is assumed sequestered, and farming and processing are assumed CO_2 neutral and the average yield is set at 1,000 tons of dry weight km^{-2} based on (135). The resulting mitigation potential projects removal of 0.0013 to 0.0027 Gt CO_2-eq yr^{-1} by 2030 and 0.0067 to 0.044 Gt CO_2-eq yr^{-1} by 2050. These values are considerably lower than those resulting from Project Drawdown's model. A thorough analysis was conducted to understand this difference. Three adjustments were made to (8)'s mitigation impact values for 2030 and 2050 to compare with Project Drawdown's values:

1. Adjustment 1: In (8) the long-term sequestration calculation includes a 24.5% carbon content in macroalgae, 60% export of DOC/POC, and 25% long-term sequestration of exported carbon vs. 28.5% carbon content, 55% export, and 32% long-term sequestration of exported carbon in our study.
2. Adjustment 2: (8) used an average yield of 10 t dry weight ha^{-1} yr^{-1} (not referenced to any particular source) vs. our study's 24.8 t dry weight ha^{-1} yr^{-1}, which was derived from the meta-analysis of 44 data points from 16 sources.
3. Adjustment 3: (8)'s adoption areas reached 0.9 and 1.6 Mha in 2030 and 4.9 and 32.4 Mha in 2050 for scenarios 1 and 2, respectively. Project Drawdown's average values for the *Plausible* and *Ambitious scenarios* resulted in 5.1 and 9.6 Mha in 2030 and 13.4 and 25.1 Mha in 2050, respectively.

After applying these three adjustments to (8)'s mitigation potential values, the resulting values are: 0.05 and 0.06 Gt CO_2-eq yr^{-1} in 2030 and 0.02 and 0.13 Gt CO_2-eq yr^{-1} in 2050, in line with Project Drawdown's *Plausible scenario* (0.06 and 0.12 Gt CO_2-eq yr^{-1} in 2030 and 2050, respectively).

The financial analysis of the solution included collecting data for the first costs of farm installation, operating costs over a respective time period, and net profit margin. First costs were based on 13 data points from 3 sources, operating costs were based on 14 data points from 4 sources, and net profit margin was based on 12 data points from 2 sources.

S6. Climate impact and uncertainty analysis

The final step of the analysis was a calculation of climate impact. Every Mha adopted between 2018 and 2060 (as specified by the *Low Adoption, Plausible*, and *Ambitious scenarios*) was multiplied by a climate variable specific for a given solution (Table 2, S5) to obtain CO_2-eq emissions of solution implementation between 2018 and 2060. These results were compared to the *Reference scenario* over the same time period, and the difference between them was the cumulative carbon avoided emissions (presented in Results) called "climate impact" (Fig. S1).

To account for the uncertainty in climate impact estimates, error propagation has been applied. This involves taking the standard deviation of the calculated averages of the main input variables and propagating that error throughout the relevant formulas to approximate the output errors of these formulas. Any value that does not have an error associated with it is referred to as "exact," which does not mean that it is accurate and precise but that there is no quantitative estimate of error associated with the measurement.

The formula for carbon removal (additional sequestration of carbon) is presented in the Methodology section in the main text**.** *NAOU* represents Net Annual Ocean Units and is used to scale the per-Ocean Unit error at the end. *Ty*, the total amount of ocean used, is a fixed value assigned to the model and does not have a set of data from which it is averaged – thus, it does not have a standard deviation.

The only components with error, therefore, are the sequestration rate q and disturbance rate δ.

Five solutions have a disturbance rate (coastal wetlands protection, coastal wetlands restoration, macroalgae protection, macroalgae restoration, and seafloor protection); for those that do not, it is assumed to be 0. In these cases, the error in the sequestration rate is simply:

$$\Delta S_y^{\mathrm{CO2}} = \Delta q$$

For those that do have disturbance rates, the error associated with sequestration is:

$$\Delta S_y^{\mathrm{CO2}} = S_y^{\mathrm{CO2}} \times \left[\left(\frac{\Delta q}{q}\right)^2 + \left(\frac{\Delta \delta}{1-\delta}\right)^2\right]^{0.5}$$

The avoided carbon emissions formula is also presented in the Methodology. Recalling that the appropriate adoption values in *NAOU* are exact, and also that they will be multiplied by the per-Ocean Area Unit error estimates, direct emissions reduction and error were estimated as follows:

$$\Delta Direct = [(\Delta EF_{con}^{OD})^2 + (\Delta EF_{con}^{OD})^2]^{0.5}$$

Figure S1 Conceptual diagram of Project Drawdown's modelling framework applied to blue-carbon solutions to climate change.

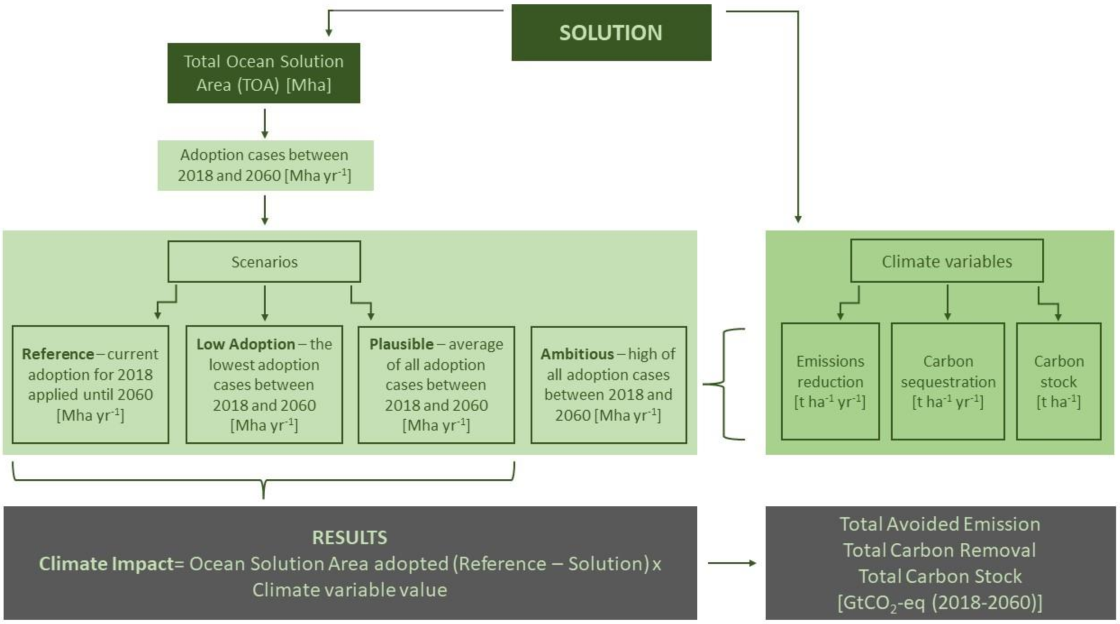

Table S1. Total Ocean Solutions Area (TOA) and current adoption for all ocean solutions.

Solution name	Max solution TOA [Mha]	Current adoption in 2018 [Mha]	Justification
Coastal wetlands protection	44.46	12.6	The TOA for this solution represents the global coverage area of mangroves, seagrasses, and saltmarshes, excluding the area subjected to degradation. Mangrove forest extent was estimated at 13.23 Mha for the year 2010 based on satellite observations (168). Seagrass meadows occupy an estimated 25.73 Mha based on average from three data points - global synthesis in the Global Distribution of Seagrasses (34.50 Mha) (169) and the most recent low (16.04 Mha) and high (26.66 Mha) estimates (170). Saltmarsh extent was estimated at 5.50 Mha (134). The annual degradation rate for mangrove forests is estimated to be 0.99% based on 4 sources (20, 69, 171, 172). For seagrasses, the annual degradation rate is estimated at 2.18% based on 3 sources (19, 23, 173). The annual loss rate of saltmarsh is estimated at 1.50% (19), which is the same rate cited by later sources.

Coastal wetlands restoration	7.32	0	The total restorable ocean-land interface area available for mangroves was set at 0.80 Mha and excludes areas converted to urban landscapes as well as “areas that have become, through erosion or inundation, non-tidal waters” (174). It should be noted that this figure is based on the loss of mangroves’ area from 1996 onwards, which means that it is a lower-bound estimate given that losses before 1996 could be restorable (174).<br><br>The total restorable area for seagrasses restoration results from (173)’s estimation of global seagrasses area loss between 1879 and 2006 (5.10 Mha).<br><br>The degraded salt marshes area resulting from the literature review is quite limited, and the TOA calculation is based on figures for the USA. The rationale is the following: the USA’s salt marshes area is 1.88 Mha, which represents 34% of the global salt marshes area (5.5 Mha) based on (134). According to (52), the loss of salt marshes area in the USA totaled 13,450 ha between 1998-2004. According to (175), the loss of salt marshes area in the USA totaled 45,140 ha between 2004-2009 (4.5 years of analysis), with less than 1% resulting from conversion to urban uses. Based on this data, four TOAs were created and their average was used as the solution TOA in the model (1.4 Mha):<br><br>• TOA 1 results from applying the yearly loss (in ha) of the USA salt marshes area from Dahl (2006) to a period of 127 years and scaling up that figure to a world level.<br><br>• TOA 2 results from applying the yearly loss (in ha) of the USA salt marshes area from Dahl (2009) to a period of 127 years and scaling up that figure to a world level.<br><br>• TOA 3 results from applying the yearly loss (in ha) of the USA salt marshes area from Dahl (2006) to a period of 30 years and scaling up that figure to a world level.<br><br>• TOA 4 results from applying the yearly loss (in ha) of the USA salt marshes area from Dahl (2009) to a period of 30 years and scaling up that figure to a world level.
Macroalgae protection	355.0 (2014)<br>354.7 (2060)	0	TOA for this solution was built based on (17)’s wild macroalgae area (355 Mha) -- excluding an estimate of the wild macroalgae harvested area (0.007 Mha yr^{-1}) up to 2060 -- and degradation rates for macroalgae forest based on the meta-analysis by (22) and other studies published after 2016 (176–187). Therefore, the TOA for this solution is not constant but changes with time.

Macroalgae restoration	156	0	The TOA for this solution was built by subtracting the total undegraded macroalgae forest area based on the Macroalgae protection TOA adoption in 2060 of *Ambitious* scenario, so it represents the unprotected wild macroalgae under ambitious cases.
Seafloor protection	490	0	The TOA represents the total seafloor of oxic sediments swept by bottom-trawling gear on an annual basis based on the most recent global estimation (10). This is often called a bottom-trawling footprint or swept area (10, 188). The trawling footprint of oxygenated sediments only has been chosen for this solution, as there is a higher potential of carbon resuspension for this type of sediment (10, 147).
Seaweed farming	240	0.2	The TOA is based on the (155) analysis of the suitable area for seaweed farming considering nutrients and temperature constraints. According to this study, 48 million km^2 (4,800 Mha) of ocean is suitable for seaweed farming. The ocean area included in the model is 240 Mha, 5% of the (155) value, which is smaller than the wild macroalgae area estimate of 355 Mha (17) but in line with 221 Mha estimated as potential macroalgae farming expansion area (189). The rationale behind choosing 5% of 4,800 Mha was setting a conservative TOA for this solution.

Table S2. Adoption cases for all ocean-based solutions.

Solution	Adoption cases	References
Coastal wetlands protection	Eight adoption cases were developed for each of the three types of coastal wetlands using a linear growth curve. Given the small area of coastal wetlands, the high urgency because of the annual degradation of unprotected coastal wetlands, and the high mitigation efficiency of protection, the adoption cases assume that 80 or 100% of the wetlands remaining in 2030 or 2050 will be protected. Another key variable governing the adoption case is the potential for change in the degradation rate over time. As discussed in S1, although direct measurements of the expansion of protection are elusive, annual rates of mangrove degradation appear to be decreasing by 30 basis points per decade. Therefore, we created an adoption case with constant degradation rates as a lower bound on adoption and an adoption case with degradation rates that decrease by 30 basis points per decade as a plausible measure of the global response to the critical importance of coastal wetlands. For each case and each coastal wetland type, the annual protection rate – figured as the annual percentage increase in the protected area – was adjusted so that the adoption targets for that case are reached.<br><br>1. Adoption case one: This adoption case assumes constant degradation rates and that 100% of the remaining coastal wetlands are protected by 2050 (annual protection rates: 3.0%, 2.2%, and 2.0%).<br>2. Adoption case two: This adoption case assumes constant degradation rates and that 80% of the remaining coastal wetlands are protected by 2050 (protection rates: 2.3%, 1.5%, and 1.3%).*<br>3. Adoption case three: This is adoption case one with the assumption that 100% of the remaining coastal wetlands are protected by 2030 (protection rates: 8.5%, 7.3%, and 7.8%).<br>4. Adoption case four: This is adoption case two with the assumption that 80% of the remaining coastal wetlands are protected by 2030 (protection rates: 6.6%, 5.4%, and 5.8%).<br>5. Adoption case five: This is adoption case one with the assumption that degradation rates will decrease by 30 basis points per	The scenarios were developed based on High Level Panel for Sustainable Ocean recommendations.

	decade (protection rates: 3.5%, 2.7%, 2.5%).<br>6. Adoption case six: This is adoption case two with the assumption that degradation rates will decrease by 30 basis points per decade (protection rates: 2.9%, 2.0%, and 1.8%).<br>7. Adoption case seven: This is adoption case three with the assumption that degradation rates will decrease by 30 basis points per decade (protection rates: 8.8%, 7.6%, and 8.0%).<br>8. Adoption case eight: This is adoption case four with the assumption that degradation rates will decrease by 30 basis points per decade (protection rates: 6.8%, 5.65%, and 6.0%).	
Coastal wetlands restoration	Six adoption cases were developed for each of the three types coastal wetlands using a linear growth curve:<br><br>1. Adoption case one: The National Mission for a Green India included a commitment to restore 0.1 Mha of mangroves in 10 years (by 2020). We assume that this commitment will be replicated in the next three decades, resulting in 300,000 additional ha being restored by 2050. This would represent a restoration of 87% of the mangrove area in India. We apply this % to the global mangrove area.<br>2. Adoption case two: Guatemala's National Determinded Contributions includes the restoration of 10,000 ha of mangroves by 2045. This would represent 38% of the total mangrove area in the country. We use the average of India (87%) and Guatemala (38%) and apply it (62%) to the TOA.*<br>3. Adoption case three: This is adoption case 1 assuming 100% of adoption is reached in 2030.<br>4. Adoption case four: This is adoption case 2 assuming 100% of adoption is reached in 2030.<br>5. Adoption case five: linear increase to 100% TOA by 2050.<br>6. Adoption case six: linear increase to 100% TOA by 2030..	The scenarios were developed based on two policies: Guatemala’s National Restoration Strategy, which aims to restore 10,000 ha of mangroves between 2015 and 2045; and the National Mission for a Green India, which included a commitment to restore 100,000 ha of mangroves between 2010 and 2020.
Macroalgae protection	Three adoption cases were developed for this solution based on projections derived from historical growth in Marine Protected Areas, as well as recent	(28, 38)

	international attention and commitment to the goal to protect 30% of the ocean by 2030. Each growth case incorporated a maximum area of the Total Ocean Area minus the degraded ocean area (Constrained TOA).<br><br>1. Adoption case one: 30% of TOA protected by 2030; an optimistic case based on full adoption of the international commitment to protect 30% of the ocean by 2030.*<br>2. Adoption case two: 30% of TOA protected by 2050, following the less optimistic assumption of the International Union for Conservation of Nature (which recommends 30% of highly protected MPA coverage by 2030).<br>3. Adoption case three: 50% of TOA protected by 2044, following projected growth rates as described in Duarte et al., 2020.	
Macroalgae restoration	Five adoption cases were developed for this solution using a combination of more conservative and more ambitious approaches. Each growth case incorporated a maximum area of the Total Ocean Area available for restoration (degraded TOA).<br><br>1. Adoption case one: this case is based on the projection of restoration area data from Korea, which has had the largest proportional adoption of macroalgae forest restoration, and assumes the same level of adoption by other regions with macroalgae forest habitats. This forms the most optimistic possible case but was excluded from model averages.<br>2. Adoption case two: this case is based on an extrapolation of global restoration project data extracted from the review study by Eger et al. 2020 from 2017 to 2019. The case is based on the cumulative size of large-scale kelp forest restoration projects, with growth projected into the future based on a best-fit 2nd-order polynomial projection.<br>3. Adoption case three: this case assumes that countries or regions accounting for 5% of the total area available for restoration achieve Korea's level of adoption.*<br>4. Adoption case four: this case assumes that countries or regions accounting for 10 % of the total available area for restoration achieve Korea's level of adoption; it projects 10% of Korea's restoration area applied to the full degraded TOA.	(61, 190)

	5. Adoption case five: This case assumes that countries or regions accounting for 50% of the total available area for restoration achieve Korea's level of adoption; it projects 50% of Korea's restoration area applied to the full degraded TOA. This is the most optimistic case that was included in model averages.	
Seafloor protection	Five adoption cases were developed for this solution using a linear growth curve. Ocean seafloor can be protected through two different mechanisms: marine reserves that prohibit any fishing activity within their boundaries, and bottom-trawling bans or closures of ocean areas. Therefore, adoption cases represent global MPA growth commitments and/or bottom-trawling bans applied in some countries:<br><br>1. Adoption case one: This case assumes 30% of TOA protected by 2030, as an optimistic assumption following IUCN (which recommends 30% highly-protected MPA coverage by 2030). After 2030, it assumes linear growth.<br>2. Adoption case two: This case assumes 30% of TOA protected by 2050, following the less optimistic assumption of IUCN (which recommend 30% highly-protected MPA coverage by 2030).*<br>3. Adoption case three: This case assumes 50% of TOA protected by 2050, based on the bottom-trawling bans of some countries.<br>4. Adoption case four: This case assumes 70% of TOA protected by 2050, based on the bottom-trawling bans of some countries.<br>5. Adoption case five: This case assumes 90% of TOA protected by 2050, based on the bottom-trawling bans of some countries.	(28, 38, 191)
Seaweed farming	Ten custom adoption cases were created using FAO macroalgae production data from 2006-2016. The annual growth rate was projected using World Bank Group (2016) and Hoegh-Guldberg et al. 2019 assumptions. In the absence of actual data on trends in the macroalgae area, the future area for this solution is projected based on the annual growth rate in production as estimated using the listed sources above:	(8, 192, 193)

	1. Adoption case one: the FAO 2006-2016 data lists country-level production of macroalgae. An annual rate of increase in production was calculated for each of the given countries. The highest increase was reported in Indonesia (9.96%). This case projects the future growth of the macroalgae area based on Indonesia's annual rate, applying it to the current adoption.<br>2. Adoption case two: the FAO 2006-2016 data lists country-level production of macroalgae. An annual rate of increase in production was calculated for each of the given countries. The second-highest increase was reported in China (3.34%). This case projects the future growth of the macroalgae area based on the China's annual rate, applying it to the current adoption.*<br>3. Adoption case three: the FAO 2006-2016 data lists country-level production of macroalgae. An annual rate of increase in production was calculated for each of the given countries. This case projects the future growth of the macroalgae area based on the global average annual rate (5.57%), applying it to the current adoption.<br>4. Adoption case four: this case projects the future growth of the macroalgae area based on the global average annual rate as reported by World Bank 2016 (14%), applying it to the current adoption.<br>5. Adoption case five: this case projects the future growth of the macroalgae area based on the annual rate of Indonesia (9.96%), applying it to the maximum TOA allocated for this solution.<br>6. Adoption case six: this case projects the future growth of the macroalgae area based on the annual rate of China (3.34%), applying it to the maximum TOA allocated for this solution.<br>7. Adoption case seven: this case projects the future growth of the macroalgae area based on the global average annual rate (5.57%), applying it to the maximum TOA allocated for this solution.<br>8. Adoption case eight: this case projects the future growth of macroalgae area based on the global average annual rate as reported by World Bank 2016 (14%), applying it to the maximum TOA allocated for this solution.	

	9. Adoption case nine: this case projects the future growth of the macroalgae area based on the production estimates given by Hoegh-Guldberg et al. 2019, using the low estimates of 8.3% annual growth rate. The case assumes a linear projection to the 2050 adoption area of Hoegh-Guldberg et al. 2019’s scenario 1.<br>10. Adoption case ten: this case projects the future growth of the macroalgae area based on the production estimates given by Hoegh-Guldberg et al. 2019, using the high estimates of 14% annual growth rate. The case assumes a linear projection to the 2050 adoption area of Hoegh-Guldberg et al. 2019’s scenario 2.	

*Adoption cases marked with asterisks were used to estimate the *Low protection scenario* as presented in Fig. 4

SI References

67. T. S. Bianchi, et al., Historical reconstruction of mangrove expansion in the Gulf of Mexico: Linking climate change with carbon sequestration in coastal wetlands. Estuarine, Coastal and Shelf Science 119, 7–16 (2013).

68. G. L. Chmura, S. C. Anisfeld, D. R. Cahoon, J. C. Lynch, Global carbon sequestration in tidal, saline wetland soils. Global biogeochemical cycles 17 (2003).

69. S. Bouillon, et al., Mangrove production and carbon sinks: A revision of global budget estimates. Global Biogeochemical Cycles 22 (2008).

70. D. M. Alongi, Carbon sequestration in mangrove forests. Carbon Management 3, 313–322 (2012).

71. J. L. Breithaupt, J. M. Smoak, T. J. Smith, C. J. Sanders, A. Hoare, Organic carbon burial rates in mangrove sediments: Strengthening the global budget. Global Biogeochemical Cycles 26 (2012).

72. J. Hutchison, A. Manica, R. Swetnam, A. Balmford, M. Spalding, Predicting Global Patterns in Mangrove Forest Biomass. Conservation Letters 7, 233–240 (2014).

73. D. C. Donato, et al., Mangroves among the most carbon-rich forests in the tropics. Nature Geoscience 4, 293–297 (2011).

74. J. Siikamaki, J. N. Sanchirico, S. L. Jardine, Global economic potential for reducing carbon dioxide emissions from mangrove loss. Proceedings of the National Academy of Sciences 109, 14369–14374 (2012).

75. C. E. Lovelock, J. W. Fourqurean, J. T. Morris, Modeled CO2 Emissions from Coastal Wetland Transitions to Other Land Uses: Tidal Marshes, Mangrove Forests, and Seagrass Beds. Front. Mar. Sci. 4 (2017).

76. F. Sidik, C. E. Lovelock, CO2 efflux from shrimp ponds in Indonesia. PloS one 8, e66329 (2013).

77. C. E. Lovelock, R. W. Ruess, I. C. Feller, CO2 efflux from cleared mangrove peat. PloS one 6, e21279 (2011).

78. D. R. Cahoon, et al., Mass tree mortality leads to mangrove peat collapse at Bay Islands, Honduras after Hurricane Mitch. Journal of ecology 91, 1093–1105 (2003).

79. D. A. Miteva, B. C. Murray, S. K. Pattanayak, Do protected areas reduce blue carbon emissions? A quasi-experimental evaluation of mangroves in Indonesia. Ecological Economics 119, 127–135 (2015).

80. J. K. S. Lang'at, et al., Rapid Losses of Surface Elevation following Tree Girdling and Cutting in Tropical Mangroves. PLOS ONE 9, e107868 (2014).

81. J. B. Kauffman, C. Heider, J. Norfolk, F. Payton, Carbon stocks of intact mangroves and carbon emissions arising from their conversion in the Dominican Republic. Ecological Applications 24, 518–527 (2014).

82. R. H. Bulmer, C. J. Lundquist, L. Schwendenmann, Sediment properties and CO2 efflux from intact and cleared temperate mangrove forests. Biogeosciences 12, 6169–6180 (2015).

83. CEC, "North American Blue Carbon Scoping Study" (Commission for Environmental Cooperation, 2013).

84. S. L. Jardine, J. V. Siikamäki, A global predictive model of carbon in mangrove soils. Environ. Res. Lett. 9, 104013 (2014).

85. S. E. Hamilton, D. A. Friess, Global carbon stocks and potential emissions due to mangrove deforestation from 2000 to 2012. Nature Climate Change 8, 240 (2018).

86. D. R. Brown, et al., Seagrass, mangrove and saltmarsh sedimentary carbon stocks in an urban estuary; Coffs Harbour, Australia. Regional Studies in Marine Science 8, 1–6 (2016).

87. R. F. Connor, G. L. Chmura, C. B. Beecher, Carbon accumulation in Bay of Fundy salt marshes: Implications for restoration of reclaimed marshes. Global Biogeochemical Cycles 15, 943–954 (2001).

88. J. C. Kathilankal, et al., Tidal influences on carbon assimilation by a salt marsh. Environmental Research Letters 3, 044010 (2008).

89. M. J. Loomis, C. B. Craft, Carbon sequestration and nutrient (nitrogen, phosphorus) accumulation in river-dominated tidal marshes, Georgia, USA. Soil Science Society of America Journal 74, 1028–1036 (2010).

90. K. Drake, H. Halifax, S. C. Adamowicz, C. Craft, Carbon Sequestration in Tidal Salt Marshes of the Northeast United States. Environmental management 56, 998–1008 (2015).

91. S. Crooks, D. Herr, J. Tamelander, D. Laffoley, J. Vandever, Mitigating climate change through restoration and management of coastal wetlands and near-shore marine ecosystems: challenges and opportunities (2011) (December 11, 2015).

92. X. Ouyang, S. Y. Lee, Updated estimates of carbon accumulation rates in coastal marsh sediments. Biogeosciences 11, 5057–5071 (2014).

93. P. I. Macreadie, et al., Carbon sequestration by Australian tidal marshes. Scientific Reports 7, 44071 (2017).

94. N.-S. Bu, et al., Reclamation of coastal salt marshes promoted carbon loss from previously-sequestered soil carbon pool. Ecological Engineering 81, 335–339 (2015).

95. T. C. Coverdale, et al., Indirect Human Impacts Reverse Centuries of Carbon Sequestration and Salt Marsh Accretion. PLOS ONE 9, e93296 (2014).

96. E. Pidgeon, "Blue Carbon: A transformational tool for marine management and conservation globally" (Consevation International, 2014).

97. R. A. Johnson, A. G. Gulick, A. B. Bolten, K. A. Bjorndal, Blue carbon stores in tropical seagrass meadows maintained under green turtle grazing. Scientific Reports 7, 13545 (2017).

98. A. I. Sousa, et al., 'Blue Carbon' and Nutrient Stocks of Salt Marshes at a Temperate Coastal Lagoon (Ria de Aveiro, Portugal). Scientific Reports 7, 41225 (2017).

99. C. M. Duarte, et al., Seagrass community metabolism: Assessing the carbon sink capacity of seagrass meadows: SEAGRASS COMMUNITY METABOLISM. Global Biogeochem. Cycles 24, n/a-n/a (2010).

100. H. Kennedy, et al., Seagrass sediments as a global carbon sink: isotopic constraints. Global Biogeochemical Cycles 24 (2010).

101. N. Marbà, et al., Impact of seagrass loss and subsequent revegetation on carbon sequestration and stocks. J Ecol 103, 296–302 (2015).

102. O. Serrano, J. J. Kelleway, C. Lovelock, P. S. Lavery, “Conservation of Blue Carbon Ecosystems for Climate Change Mitigation and Adaptation” in Coastal Wetlands, (Elsevier, 2019), pp. 965–996.

103. J. T. Greiner, K. J. McGlathery, J. Gunnell, B. A. McKee, Seagrass Restoration Enhances “Blue Carbon” Sequestration in Coastal Waters. PLoS ONE 8, e72469 (2013).

104. J. W. Fourqurean, et al., Seagrass ecosystems as a globally significant carbon stock. Nature Geoscience 5, 505–509 (2012).

105. D. Laffoley, G. D. Grimsditch, The management of natural coastal carbon sinks (Iucn, 2009) (December 11, 2015).

106. E. C. Brevik, J. A. Homburg, A 5000 year record of carbon sequestration from a coastal lagoon and wetland complex, Southern California, USA. Catena 57, 221–232 (2004).

107. K. L. McKee, D. R. Cahoon, I. C. Feller, Caribbean mangroves adjust to rising sea level through biotic controls on change in soil elevation. Global Ecology and Biogeography 16, 545–556 (2007).

108. A. M. Nahlik, M. S. Fennessy, Carbon storage in US wetlands. Nature Communications 7, 13835 (2016).

109. B. W. Griscom, et al., Natural climate solutions. PNAS 114, 11645–11650 (2017).

110. S. G. Salmo, C. Lovelock, N. C. Duke, Vegetation and soil characteristics as indicators of restoration trajectories in restored mangroves. Hydrobiologia 720, 1–18 (2013).

111. A. C. Ferreira, G. Ganade, J. Luiz de Attayde, Restoration versus natural regeneration in a neotropical mangrove: Effects on plant biomass and crab communities. Ocean & Coastal Management 110, 38–45 (2015).

112. A. C. Ferreira, L. E. A. Bezerra, H. Matthews-Cascon, Aboveground carbon stock in a restored neotropical mangrove: influence of management and brachyuran crab assemblage. Wetlands Ecol Manage 27, 223–242 (2019).

113. N. Matsui, K. Morimune, W. Meepol, J. Chukwamdee, Ten year evaluation of carbon stock in mangrove plantation reforested from an abandoned shrimp pond. Forests 3, 431–444 (2012).

114. F. Sidik, M. Fernanda Adame, C. E. Lovelock, Carbon sequestration and fluxes of restored mangroves in abandoned aquaculture ponds. Journal of the Indian Ocean Region, 1–16 (2019).

115. S. Aksornkoae, Reforestacioon de bosques de manglar en Tailandia. In: Field, C. (Ed.), (International Tropical Timber Organization and International Society for Mangrove Ecosystems, 1996).

116. Y. M. Thant, M. Kanzaki, S. Ohta, M. M. Than, Carbon sequestration by mangrove plantations and a natural regeneration stand in the Ayeyarwady Delta, Myanmar. Tropics 21, 1–10 (2012).

117. A. G. DelVecchia, et al., Organic carbon inventories in natural and restored Ecuadorian mangrove forests. PeerJ 2, e388 (2014).

118. J. G. Kairo, J. K. S. Lang’at, F. Dahdouh-Guebas, J. Bosire, M. Karachi, Structural development and productivity of replanted mangrove plantations in Kenya. Forest Ecology and Management 255, 2670–2677 (2008).

119. L. D. Camacho, et al., Tree biomass and carbon stock of a community-managed mangrove forest in Bohol, Philippines. Forest Science and Technology 7, 161–167 (2011).

120. V. N. Nam, S. D. Sasmito, D. Murdiyarso, J. Purbopuspito, R. A. MacKenzie, Carbon stocks in artificially and naturally regenerated mangrove ecosystems in the Mekong Delta. Wetlands Ecol Manage 24, 231–244 (2016).

121. F. E. Putz, H. T. Chan, Tree growth, dynamics, and productivity in a mature mangrove forest in Malaysia. Forest Ecology and Management 17, 211–230 (1986).

122. Mackenzie, Richard A., et al., Sedimentation and belowground carbon accumulation rates in mangrove forests that differ in diversity and land use: a tale of two mangroves. Wetlands Ecol Manage 24, 245–261 (2016).

123. IPCC, “2013 Supplement to the 2006 IPCC Guidelines for National Greenhouse Gas Inventories: Wetlands, Hiraishi, T., Krug, T., Tanabe, K., Srivastava, N., Baasansuren, J., Fukuda, M. and Troxler, T.G. (eds).” (IPCC).

124. A. Burden, A. Garbutt, C. D. Evans, Effect of restoration on saltmarsh carbon accumulation in Eastern England. Biol. Lett. 15, 20180773 (2019).

125. F. Artigas, et al., Long term carbon storage potential and CO 2 sink strength of a restored salt marsh in New Jersey. Agricultural and Forest Meteorology 200, 313–321 (2015).

126. J. C. Callaway, E. L. Borgnis, R. E. Turner, C. S. Milan, Carbon Sequestration and Sediment Accretion in San Francisco Bay Tidal Wetlands. Estuaries and Coasts 35, 1163–1181 (2012).

127. A. Burden, R. A. Garbutt, C. D. Evans, D. L. Jones, D. M. Cooper, Carbon sequestration and biogeochemical cycling in a saltmarsh subject to coastal managed realignment. Estuarine, Coastal and Shelf Science 120, 12–20 (2013).

128. C. A. Adams, J. E. Andrews, T. Jickells, Nitrous oxide and methane fluxes vs. carbon, nitrogen and phosphorous burial in new intertidal and saltmarsh sediments. Science of The Total Environment 434, 240–251 (2012).

129. A. Thorhaug, H. M. Poulos, J. López-Portillo, T. C. W. Ku, G. P. Berlyn, Seagrass blue carbon dynamics in the Gulf of Mexico: Stocks, losses from anthropogenic disturbance, and gains through seagrass restoration. Science of The Total Environment 605–606, 626–636 (2017).

130. C. Mcowen, et al., A global map of saltmarshes. Biodiversity Data Journal 5, e11764 (2017).

131. C. M. Duarte, Reviews and syntheses: Hidden forests, the role of vegetated coastal habitats in the ocean carbon budget. Biogeosciences 14, 301–310 (2017).

132. S. van de Velde, V. Van Lancker, S. Hidalgo-Martinez, W. M. Berelson, F. J. R. Meysman, Anthropogenic disturbance keeps the coastal seafloor biogeochemistry in a transient state. Sci Rep 8 (2018).

133. QGIS.org, QGIS Geographic Information System.Open Source Geospatial Foundation Project. (2020).

134. Flanders Marine Institute, Maritime Boundaries Geodatabase: Maritime Boundaries and Exclusive Economic Zones (200NM), version 11. (2019).

135. J. Depestele, et al., Comparison of mechanical disturbance in soft sediments due to tickler-chain SumWing trawl vs. electro-fitted PulseWing trawl. ICES J Mar Sci 76, 312–329 (2019).

136. J. Depestele, et al., Measuring and assessing the physical impact of beam trawling. ICES J Mar Sci 73, i15–i26 (2016).

137. J. Martín, P. Puig, A. Palanques, A. Giamportone, Commercial bottom trawling as a driver of sediment dynamics and deep seascape evolution in the Anthropocene. Anthropocene 7, 1–15 (2014).

138. J. B. Jones, Environmental impact of trawling on the seabed: A review. New Zealand Journal of Marine and Freshwater Research 26, 59–67 (1992).

139. D. E. Duplisea, S. Jennings, S. J. Malcolm, R. Parker, D. B. Sivyer, Modelling potential impacts of bottom trawl fisheries on soft sediment biogeochemistry in the North Sea†. Geochemical Transactions 2, 112 (2001).

140. C. Yu, Z. Chen, L. Chen, P. He, The rise and fall of electrical beam trawling for shrimp in the East China Sea: technology, fishery, and conservation implications. ICES J Mar Sci 64, 1592–1597 (2007).

141. C. S. Rose, J. Gauvin, C. Hammond, Effective herding of flatfish by cables with minimal seafloor contact. undefined (2010) (September 24, 2020).

142. J. G. Hiddink, et al., Global analysis of depletion and recovery of seabed biota after bottom trawling disturbance. PNAS 114, 8301–8306 (2017).

143. T. Luisetti, et al., Quantifying and valuing carbon flows and stores in coastal and shelf ecosystems in the UK. Ecosystem Services 35, 67–76 (2019).

144. S. Paradis, et al., Organic matter contents and degradation in a highly trawled area during fresh particle inputs (Gulf of Castellammare, southwestern Mediterranean). Biogeosciences 16, 4307–4320 (2019).

145. A. Pusceddu, et al., Chronic and intensive bottom trawling impairs deep-sea biodiversity and ecosystem functioning. PNAS 111, 8861–8866 (2014).

146. P. Puig, et al., Ploughing the deep sea floor. Nature 489, 286–289 (2012).

147. M. Sciberras, et al., Impacts of bottom fishing on the sediment infaunal community and biogeochemistry of cohesive and non-cohesive sediments. Limnology and Oceanography 61, 2076–2089 (2016).

148. H. Zhongqi, W. Fengchang, Labile Organic Matter—Chemical Compositions, Function, and Significance in Soil and the Environment (Soil Science Society of America, Inc., 2015).

149. R. Danovaro, M. Fabiano, N. Della Croce, Labile organic matter and microbial biomasses in deep-sea sediments (Eastern Mediterranean Sea). Deep Sea Research Part I: Oceanographic Research Papers 40, 953–965 (1993).

150. R. G. Keil, D. B. Montluçon, F. G. Prahl, J. I. Hedges, Sorptive preservation of labile organic matter in marine sediments. Nature 370, 549–552 (1994).

151. H. E. Froehlich, J. C. Afflerbach, M. Frazier, B. S. Halpern, Blue Growth Potential to Mitigate Climate Change through Seaweed Offsetting. Current Biology 29, 3087-3093.e3 (2019).

152. Y. Zhang, et al., Carbon sequestration processes and mechanisms in coastal mariculture environments in China. Sci. China Earth Sci. 60, 2097–2107 (2017).

153. Sustainable Energy Ireland, “A Review of the Potential of Marine Algae as a Source of Biofuel in Ireland” (2009).

154. J. Forster, R. Radulovich, “Seaweed and food security” in Seaweed Sustainability, (Elsevier, 2015), pp. 289–313.

155. E. K. Hwang, N. Yotsukura, S. J. Pang, L. Su, T. F. Shan, Seaweed breeding programs and progress in eastern Asian countries. Phycologia 58, 484–495 (2019).

156. L. Hasselström, et al., Socioeconomic prospects of a seaweed bioeconomy in Sweden. Sci Rep 10, 1610 (2020).

157. S. Kraan, Undaria marching on; late arrival in the Republic of Ireland. J Appl Phycol 29, 1107–1114 (2017).

158. M. D. Hanisak, CULTIVATION OF GRACILARIA AND OTHER MACROALGAE IN FLORIDA FOR ENERGY PRODUCTION. 29.

159. C. Peteiro, N. Sánchez, C. Dueñas-Liaño, B. Martínez, Open-sea cultivation by transplanting young fronds of the kelp Saccharina latissima. J Appl Phycol 26, 519–528 (2014).

160. M. Ganesan, et al., Seaweed resources in India – current status of diversity and cultivation: prospects and challenges. Botanica Marina 62, 463–482 (2019).

161. Y. Zheng, R. Jin, X. Zhang, Q. Wang, J. Wu, The considerable environmental benefits of seaweed aquaculture in China. Stoch Environ Res Risk Assess 33, 1203–1221 (2019).

162. D. Valderrama, J. Cai, N. Hishamunda, N. B. Ridler, Food and Agriculture Organization of the United Nations, Eds., Social and economic dimensions of carrageenan seaweed farming (Food & Agriculture Organization of the United Nations, 2013).

163. A. D. Hughes, et al., Does seaweed offer a solution for bioenergy with biological carbon capture and storage? Greenhouse Gases: Science and Technology 2, 402–407 (2012).

164. P. Bunting, et al., The Global Mangrove Watch—A New 2010 Global Baseline of Mangrove Extent. Remote Sensing 10, 1669 (2018).

165. UNEP-WCMC, F. T. Short, Global Distribution of Seagrasses (2018) (December 11, 2018).

166. L. J. McKenzie, et al., The global distribution of seagrass meadows. Environ. Res. Lett. 15, 074041 (2020).

167. S. E. Hamilton, D. Casey, Creation of a high spatio-temporal resolution global database of continuous mangrove forest cover for the 21st century (CGMFC-21). Global Ecology and Biogeography 25, 729–738 (2016).

168. A. Strong, S. Minnemeyer, Satellite Data Reveals State of the World’s Mangrove Forests | World Resources Institute (2015) (April 11, 2019).

169. M. Waycott, et al., Accelerating loss of seagrasses across the globe threatens coastal ecosystems. Proceedings of the National Academy of Sciences 106, 12377–12381 (2009).

170. T. Worthington, M. Spalding, Mangrove Restoration Potential: a global map highlighting a critical opportunity. 36 (2018).

171. T. E. Dahl, “Status and trends of wetlands in the conterminous United States 2004 to 2009” (US Department of the Interior, US Fish and Wildlife Service, Fisheries and Habitat Conservation., 2011).

172. P. E. Carnell, M. J. Keough, Reconstructing Historical Marine Populations Reveals Major Decline of a Kelp Forest Ecosystem in Australia. Estuaries and Coasts 42, 765–778 (2019).

173. P. Casado-Amezúa, et al., Distributional shifts of canopy-forming seaweeds from the Atlantic coast of Southern Europe. Biodivers Conserv 28, 1151–1172 (2019).

174. S. D. Connell, et al., Recovering a lost baseline: missing kelp forests from a metropolitan coast. Marine Ecology Progress Series 360, 63–72 (2008).

175. B. K. Eriksson, G. Johansson, P. Snoeijs, Long-Term Changes in the Macroalgal Vegetation of the Inner Gullmar Fjord, Swedish Skagerrak Coast1. Journal of Phycology 38, 284–296 (2002).

176. K. Filbee-Dexter, C. Feehan, R. Scheibling, Large-scale degradation of a kelp ecosystem in an ocean warming hotspot. Marine Ecology Progress Series 543 (2016).

177. A. M. Friedlander, et al., Kelp forests at the end of the earth: 45 years later. PLOS ONE 15, e0229259 (2020).

178. C. R. Johnson, et al., Climate change cascades: Shifts in oceanography, species’ ranges and subtidal marine community dynamics in eastern Tasmania. Journal of Experimental Marine Biology and Ecology 400, 17–32 (2011).

179. A. Middelboe, K. Sand-Jensen, Long-term changes in macroalgal communities in a Danish estuary. Phycologia 39, 245–257 (2000).

180. L. Rogers-Bennett, C. A. Catton, Marine heat wave and multiple stressors tip bull kelp forest to sea urchin barrens. Scientific Reports 9, 15050 (2019).

181. S. E. Voerman, E. Llera, J. M. Rico, Climate driven changes in subtidal kelp forest communities in NW Spain. Marine Environmental Research 90, 119–127 (2013).

182. H. Vogt, W. Schramm, Conspicuous decline of Fucus in Kiel Bay (Western Baltic): what are the causes? Marine Ecology Progress Series 69, 189–194 (1991).

183. T. Wernberg, et al., Climate-driven regime shift of a temperate marine ecosystem. Science 353, 169–172 (2016).

184. R. O. Amoroso, et al., Bottom trawl fishing footprints on the world’s continental shelves. PNAS 115, E10275–E10282 (2018).

185. BFI, “Accelerating Blue Carbon” (Buckminster Fuller Institute, 2020).

186. E. K. Hwang, H. G. Choi, J. K. Kim, Seaweed resources of Korea. Botanica Marina 63, 395–405 (2020).

187. United Nations, “The Impacts of Fishing on Vulnerable Marine Ecosystems: Actions taken by States and regional fisheries management organizations and arrangements to give effect to paragraphs 66 to 69 of General Assembly resolution 59/25 on sustainable fisheries, regarding the impacts of fishing on vulnerable marine ecosystems” (United Nations General Assembly, 2006).

188. World Bank Group, Seaweed Aquaculture for Food Security, Income Generation and Environmental Health in Tropical Developing Countries (World Bank, Washington, DC, 2016) https:/doi.org/10.1596/24919 (February 15, 2021).

189. FAO, The State of World Fisheries and Aquaculture 2016 (SOFIA) (2016).
